# Membrane Vesicles from *Lacticaseibacillus Casei* BL23 Exhibit Antimicrobial Activity Against *Escherichia coli* and Immunostimulatory Effects on Human Peripheral Blood Mononuclear Cells

**DOI:** 10.1002/adhm.202500548

**Published:** 2025-12-05

**Authors:** Cecilia L. D'Antoni, Leila Pourtalebi Jahromi, Lorenzo Sana, Ana Paula Domínguez Rubio, Maja Dorfner, Jennifer Munkert, Heike Danzer, Philipp Arnold, Mikhail Lebedev, Esther Zanin, Oscar E. Pérez, Gregor Fuhrmann

**Affiliations:** ^1^ Departamento De Química Biológica De La Facultad de Ciencias Exactas y Naturales Universidad De Buenos Aires Buenos Aires Argentina; ^2^ Instituto De Química Biológica De La Facultad de Ciencias Exactas y Naturales Universidad De Buenos Aires Consejo Nacional de Investigaciones Científicas y Técnicas Buenos Aires Argentina; ^3^ Department of Biology Pharmaceutical Biology Friedrich‐Alexander‐Universität Erlangen‐Nürnberg (FAU) Erlangen Germany; ^4^ FAU NeW Friedrich‐Alexander‐Universität Erlangen‐Nürnberg (FAU) Erlangen Germany; ^5^ Deutsches Zentrum Immuntherapie (DZI) and Department of Internal Medicine 3 Rheumatology and Immunology Friedrich‐Alexander‐Universiät Erlangen‐Nürnberg (FAU) and Universitätsklinikum Erlangen Erlangen Germany; ^6^ Institute of Functional and Clinical Anatomy Friedrich‐Alexander‐University Erlangen‐Nürnberg (FAU) Erlangen Germany; ^7^ Department of Biology Friedrich‐Alexander‐Universität Erlangen‐Nürnberg (FAU) Erlangen Germany

**Keywords:** antimicrobial activity, immune response, membrane vesicles, probiotics

## Abstract

Probiotics are live microorganisms known for their health benefits; however, they may pose risks for immunocompromised individuals. This study explores the potential of postbiotics, specifically membrane vesicles (MVs) derived from *Lacticaseibacillus casei* BL23, as a safer alternative. MVs are isolated via ultracentrifugation (UC) followed by size exclusion chromatography (SEC), revealing a mean size of 178 nm and a concentration of 2.53×10^11^ particles mL^−1^, with moderate stability over four months at 4°C. Antimicrobial assays demonstrate that SEC‐isolated MVs effectively inhibit the growth of *Escherichia coli* DH5α. Confocal laser scanning microscopy confirms the internalization of MVs by both *E. coli* and macrophages. Additionally, MV treatment elicits a significant immune response, marked by increased levels of pro‐inflammatory cytokines (TNF‐α, IL‐6, IL‐8, IL‐1β) alongside IL‐10 production in peripheral blood mononuclear cells. Importantly, cytotoxicity assays indicate that MVs are non‐toxic to these cells and preserve epithelial barrier integrity in Caco‐2 cell monolayers. MVs showed a trend towards extending lifespan in the *Caenorhabditis elegans* in vivo model upon infection with *Pseudomonas aeruginosa*. These findings underscore the dual‐activity potential of *L. casei* BL23 MVs as effective tools for combating infections while enhancing immune function, offering a promising strategy to address antibiotic resistance.

## Graphical abstract

Membrane vesicles derived from the probiotic *Lacticaseibacillus casei* BL23 demonstrate antimicrobial properties against *Escherichia coli* and a potential biological effect in improving the overall survival of *C. elegans* infected with *Pseudomonas aeruginosa*. These vesicles stimulated immune responses in primary cells without causing toxicity. Our results indicate that probiotic vesicles have a dual effect by enhancing the immune function while combating infections, presenting a promising strategy to address concerns over antibiotic resistance while minimizing risks for vulnerable populations.

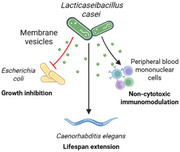

## 1 Introduction

Probiotics are living microorganisms that provide health benefits when administered in adequate amounts [1]. These microorganisms are recognized for their ability to promote gut health, enhance immune responses, and serve as alternative treatments for various health disorders; however, they also carry risks, particularly in immunocompromised populations where adverse effects can occur, including infections and antibiotic resistance [2]. There is growing evidence that non‐viable microorganisms and their bioactive compounds, known as postbiotics, can also confer health benefits [3]. In this line, extracellular vesicles (EVs) secreted by probiotics are emerging as a new category of postbiotics [4].

EVs are nanosized structures composed of a lipid bilayer containing a variety of small and macromolecules, ranging from secondary metabolites to nucleic acids and proteins. They are released by various organisms, including archaea, bacteria, and eukaryotes, as a means of intercellular communication and are named accordingly. Specifically, EVs secreted by Gram‐positive bacteria are referred to as membrane vesicles (MVs). These nano‐sized structures play a crucial role in intercellular interactions, facilitating the transfer of proteins, nucleic acids, and other biomolecules between cells [5].

Understanding the biology of MVs can enhance their application as nanopharmaceuticals [6], offering potential therapeutic and prophylactic strategies for various diseases. MVs utilize their natural ability to deliver bioactive compounds to target cells effectively and may protect their cargo from proteases and inactivation molecules normally present in the intestine [6]. Their ability to cross epithelial barriers has been demonstrated in studies such as the transcytosis of *Bacillus subtilis* MVs through an in vitro intestinal epithelial cell model [7] and opens up new possibilities for targeted therapies and drug delivery systems.

Research on the antimicrobial potential of probiotic‐derived MVs is in its early stages, with many aspects yet to be fully explored and understood. A few studies demonstrated lactobacilli MVs may combat infections through mechanisms similar to those of their parent cells, either through antimicrobial agents or through the competitive exclusion of pathogens by blocking the adhesion sites of the host tissues. For instance, MVs produced by *Lactobacillus acidophilus* ATCC 53544 can deliver bacteriocins and kill *Lactobacillus delbrueckii* when treated with lactacin B‐inducing peptide [8]. Additionally, L*actobacillus crispatus* and *Lactobacillus gasseri* MVs demonstrated the ability to reduce the attachment of gp120 HIV‐1 protein to host target cells [9], while *Lactiplantibacillus plantarum* MVs showed efficacy in preventing skin inflammation in a murine model of *Staphylococcus aureus* EV‐induced atopic dermatitis [10]. In a more recent study, *Ligilactobacillus*‐derived MVs have been shown to inhibit the growth and virulence of enteric pathogens such as *Campylobacter jejuni* and *Salmonella*, suggesting their potential as therapeutic agents in combating gastrointestinal infections [11].

*Lacticaseibacillus casei* BL23 is a well‐studied probiotic strain known for its immunomodulatory effects, as evidenced in infection and inflammation models [12, 13]. Domínguez Rubio et al. (2017) first described MVs isolated from *L. casei* BL23 using ultracentrifugation, providing initial insights into their properties and functions [14]. In their work, proteomic analysis identified a total of 103 proteins in *L. casei* BL23 MVs, including 13 proteins uniquely present in the MVs and probiotic‐associated proteins such as p40 and p75 [14]. Meanwhile, ζ potential measurement revealed a negative value of ‐8.7 mV, comparable to that reported for other Gram‐positive bacteria, suggesting colloidal stability [14]. While previous research has demonstrated the role of *L. casei* BL23 MVs against biofilm formation [15] and modest proinflammatory effects on intestinal epithelial cells [16], the overall picture of their antimicrobial activity and influence on host‐microbe interactions remains to be fully elucidated [15].

Our study aims to comprehensively characterize *L. casei* BL23 MVs isolated by ultracentrifugation followed by size exclusion chromatography (SEC), a method that offers improved purity compared to traditional ultracentrifugation techniques. We investigate these MVs as fundamental mediators of microbe‐microbe and microbe‐host communication, with an emphasis on their promising applications as biomaterials [17]. Notably, this includes the examination of their antimicrobial properties and their involvement in interspecies and interkingdom communication. Given that MVs possess the capacity to traverse biological barriers, like the intestinal epithelium, and interact with diverse cell populations, we employ multiple cell lines — including macrophages and peripheral blood mononuclear cells (PBMCs) — to study MV‐host interactions across varied biological contexts [7, 18, 19]. Our screening strategy thus aims to explore broad biological functions rather than detailed mechanistic insights. To enhance our understanding of these effects, we complement our in vitro experiments with in vivo assays utilizing *Caenorhabditis elegans*, a free‐living nematode widely recognized as a valuable model organism in biological research, with a specific focus on evaluating potential lifespan extension. Our study presents a novel approach by combining the antimicrobial and immunomodulatory properties of MVs from *L. casei* BL23, highlighting their potential as versatile biomaterials [17].

## 2 Results and Discussion

### 2.1 Characterization of *L. casei* MVs

To determine the effectiveness of SEC in isolating MVs while minimizing contamination from soluble proteins, we first analyzed the protein content in various fractions obtained through SEC, which was performed after an ultracentrifugation step. The results indicated that fraction 6 was enriched in MVs, with a protein concentration of 271 ± 75 µg mL^−1^ (*n* = 12). This measurement was crucial for confirming the efficacy of the purification technique, by ensuring that we had successfully separated the MVs from contaminant proteins, as evidenced by the clear separation of peaks observed (Figure S1A). Scalable alternatives to SEC, such as tangential flow filtration, exist and have been shown to offer efficient, reproducible, and scalable purification of EVs, which could be considered for future applications [20].

Transmission electron microscopy (TEM) analysis further supported these findings; the images of MVs separated by SEC showed well‐defined, spherical vesicles with a relatively clean background, indicating low contamination, whereas TEM images of the UC pellet prior to SEC revealed a heterogeneous background with substantial particulate contamination, which significantly impaired the clear visualization of VEs (Figure S1B).

Following this, we employed nanoparticle tracking analysis (NTA) to assess the size distribution and particle concentration of the purified MVs. The NTA results revealed that the MVs had a mean size of 178 ± 11 nm (*n* = 7) and a particle concentration of 2.53 × 10^11^ ± 0.88 × 10^11^ particles mL^−1^ (*n* = 4) (Figure S1C). These parameters are essential for understanding the characteristics of MVs, as size and concentration can significantly influence their biological activity and potential applications [21].

Interestingly, the measured size of these MVs was larger than that reported in previous studies, which utilized microscopy and dynamic light scattering techniques [14, 16]. This discrepancy may be attributed to the different measurement methods, as well as potential aggregation during the concentration step or inherent biological variability. It is important to note that NTA measures the hydrodynamic size of particles, which can be influenced by factors such as shape, surface properties, and interactions with the surrounding medium [22].

To further evaluate the integrity of MVs for potential therapeutic and biomedical applications, we monitored their long‐term stability at 4°C. Notably, we observed a decrease in particle concentration by approximately 24%, a reduction in protein concentration by about 12% and an increase in the hydrodynamic diameter of the MVs by 9% (Figure S1D) within four months of storage (*p* > 0.05). This increase in size could be attributed to adsorption of molecules onto the vesicle surface during storage [23]. These findings indicate that while some loss occurs over time, a significant proportion of their integrity is maintained. Biofunctional changes at other storage temperatures were not assessed. However, it is noted that storage at 25°C and 37°C is not recommended for protein‐based bacterial vesicles, consistent with pharmaceutical guidelines and previous reports [24, 25].

Overall, measuring protein concentrations in SEC fractions and subsequently analyzing MVs using NTA provided comprehensive insights into both the purity and stability of *L. casei* MVs.

### 2.2 Antimicrobial Assays

**FIGURE 1:**
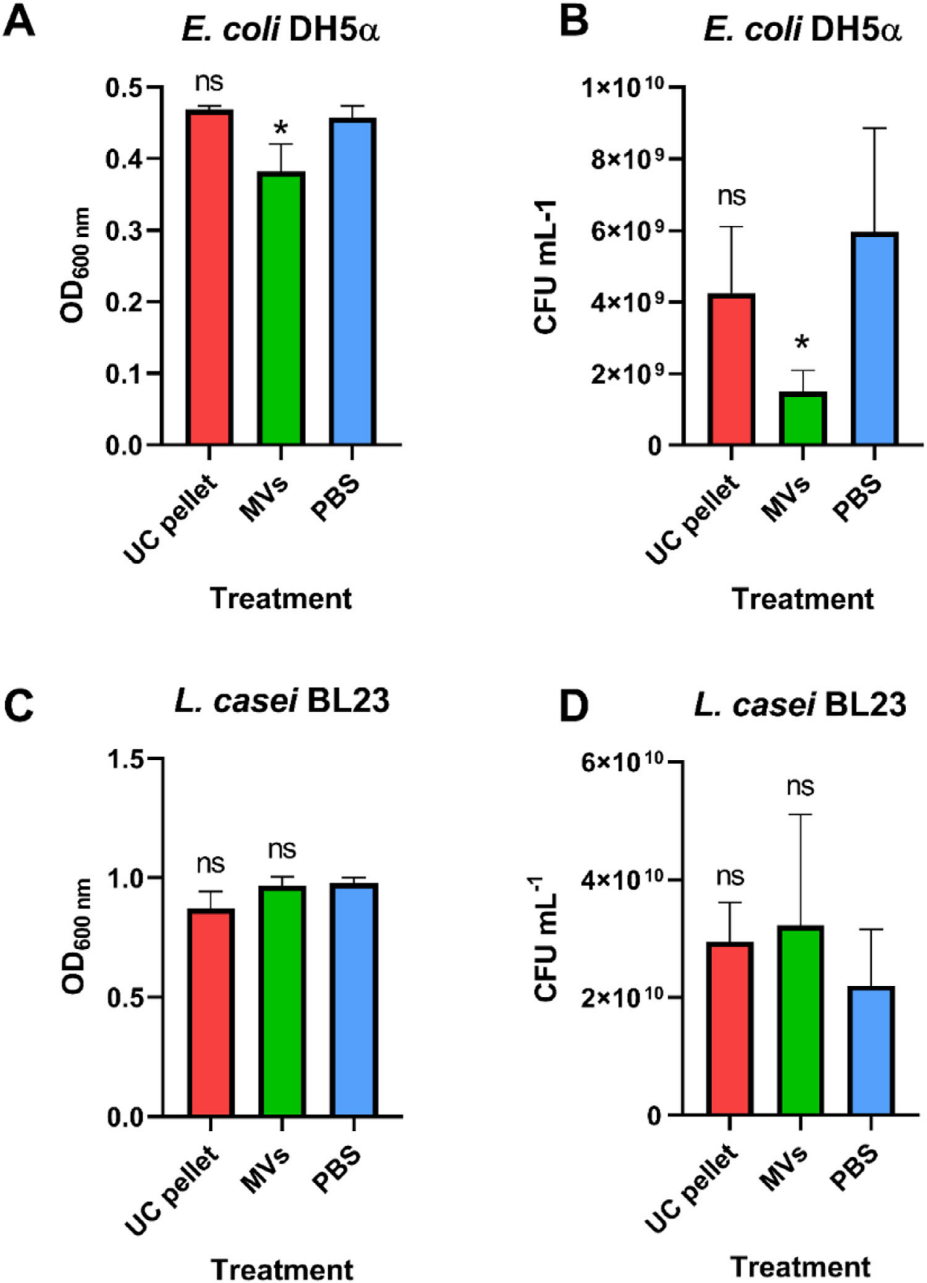
(A) Optical density (OD) of *E. coli* culture incubated for 15 h with *L. casei* BL23 ultracentrifugation pellet (UC pellet) (2.5 × 10^12^ particles mL^−1^) and membrane vesicles (MVs) (5 × 10^11^ particles mL^−1^). Phosphate buffer saline (PBS) was used as a control. Bars represent mean value and standard deviation (*n* = 4). *Significant difference compared to the control (PBS: Phosphate buffer saline) (*p* = 0.0456; one‐way ANOVA followed by Tukey comparisons). (B) Optical density (OD) of *L. casei* culture incubated for 18 h with *L. casei* BL23 ultracentrifugation pellet (UC pellet) (2.5 × 10^12^ particles mL^−1^) and membrane vesicles (MVs) (5 × 10^11^ particles mL^−1^). Phosphate buffer saline (PBS) was used as a control. Bars represent mean value and standard deviation (*n* = 3). (C) Colony forming units per mL (CFU mL^−1^) of *E. coli* incubated for 20 h with *L. casei* BL23 ultracentrifugation pellet (UC pellet) (5 × 10^11^ particles mL^−1^) and membrane vesicles (MVs) (5 × 10^11^ particles mL^−1^). The graph displays the mean value with corresponding standard errors for each treatment group. Statistical analysis was performed using log‐transformed data and the inherent variability between replicate measurements was considered (*n* = 3). *Significant difference compared to the control (p = 0.0425; two‐way ANOVA followed by Tukey comparisons). (D) Colony forming units per mL (CFU mL^−1^) of *L. casei* incubated for 20 h with *L. casei* BL23 ultracentrifugation pellet (UC pellet) (5 × 10^11^ particles mL^−1^) and membrane vesicles (MVs) (5 × 10^11^ particles mL^−1^). The graph displays the mean value with corresponding standard errors for each treatment group. Statistical analysis was performed using log‐transformed data (*n* = 3).

To evaluate *L. casei* MVs’ potential as natural antimicrobial agents inhibiting pathogenic bacteria while preserving beneficial strains within the microbiome, we determined the antimicrobial effects of the purified *L. casei* MVs and the pre‐purification ultracentrifugation pellet (UC pellet) against *E. coli* and *L. casei*. As shown in Figure 1, the UC pellet (not purified by SEC) at 2.5 × 10^12^ particles mL^−1^ did not affect the growth of *E. coli*. Interestingly, MVs (5 × 10^11^ particles mL^−1^) affected *E. coli* growth after 15 h incubation (*p *< 0.05) (Figure 1A). We further evaluated the effects of the UC pellet and MVs at the same particle concentration (5 × 10^11^ particles mL^−1^) using plate count analysis (colony forming units mL^−1^) and confirmed the inhibitory effect of MVs on *E. coli *growth (Figure 1B). These findings highlight that purifying MVs by SEC, despite increasing preparation costs and lowering yield, results in MVs exhibiting antibacterial activity absent in the low‐purity UC pellet. The MV sample may contain a higher concentration of active agents than the UC pellet, which could explain the observed inhibition of *E. coli* growth. Alternatively, the SEC purification process might remove growth‐promoting proteins from the UC pellet, potentially eliminating essential nutrients or amino acids that support *E. coli* growth dynamics. Our results highlight, that discrepancies among the functional properties of certain type of extracellular vesicles can arise due to variations in the methods used for isolating and purifying them, which affects the yield and composition of the final samples [26].

In contrast, the growth of *L. casei* was not affected by either of the treatments (UC pellet or *L. casei* MVs) (Figure 1C,D). The selective inhibitory effect of *L. casei* MVs on the growth of *E. coli* implies potential for their use as specific antimicrobial agents. Nevertheless, only a few of the previous studies report antibacterial effects of lactobacilli MVs [8, 15]. The antibacterial effects of lactobacilli MVs were previously attributed to the presence of peptidoglycan hydrolases, for example p40 and p75, the most abundant proteins in *L. casei* BL23 MVs [14, 16], which were effective against biofilm formation [15], as well as bacteriocins that target competing bacteria in the same niche [8]. Proteomic analyses of *L. casei* BL23 MVs have been conducted previously, revealing the presence of proteins with reported antibacterial properties in the literature [14]. Among them are pyruvate oxidase, involved in interspecies inhibition of certain pathogens [27], serine‐type D‐Ala‐D‐Ala carboxypeptidase, reported to inhibit bacterial quorum sensing [28], and N‐acetylmuramoyl‐L‐alanine amidase (a peptidoglycan hydrolase), which has shown antibacterial activity against various bacteria including *E. coli* DH5α [29]. The latter hydrolyzes the peptidoglycan in the bacterial cell wall, specifically the amide bond between the lactyl group of muramic acid and the α‐amino group of the L‐alanine residue [29]. Although the antibacterial activity of these enzymes has not yet been experimentally confirmed in *L. casei* BL23, these findings underscore their potential and justify further investigation.

**FIGURE 2:**
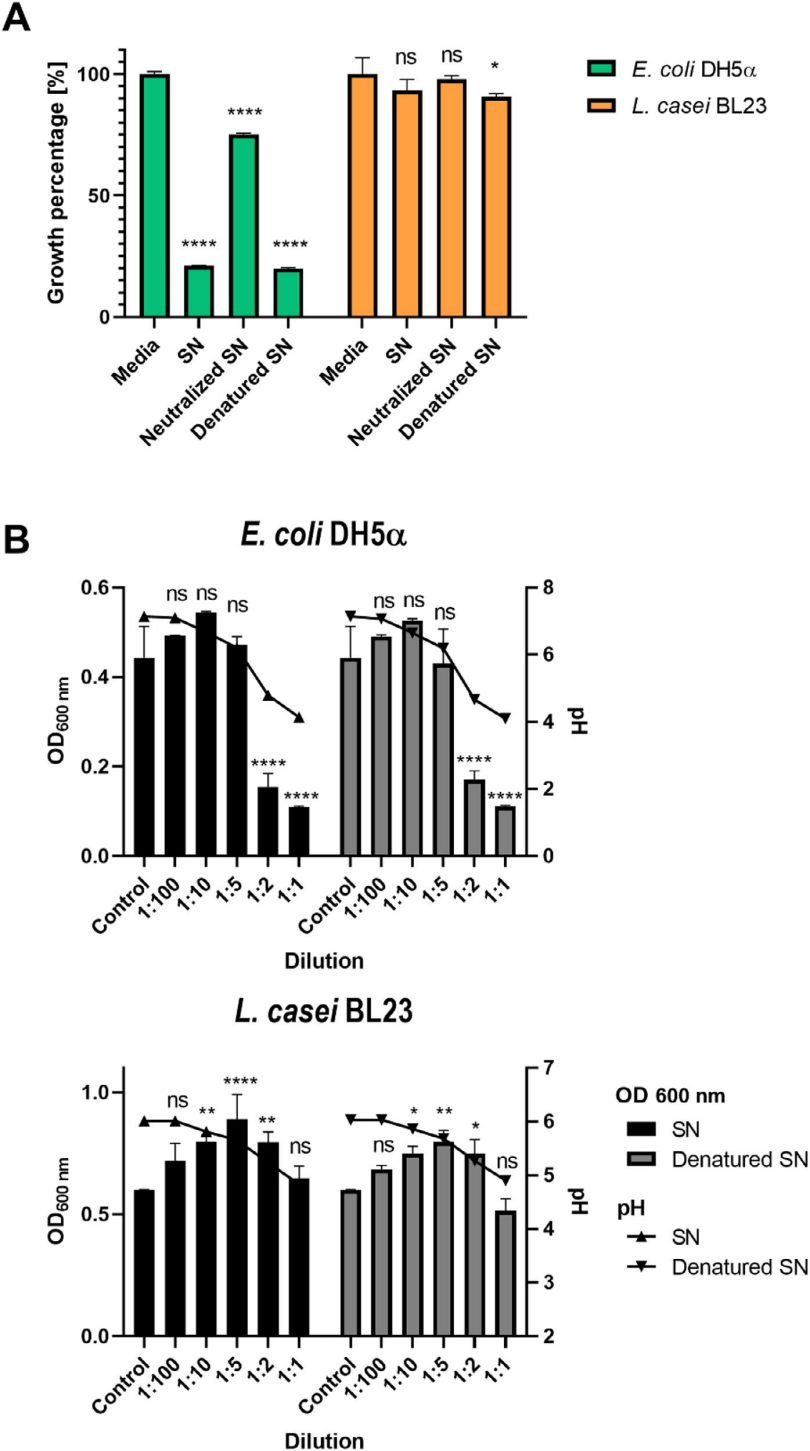
(A) Percentage of growth of *L. casei* culture (orange) and *E. coli* culture (green) incubated until the stationary phase of growth with *L. casei* ultracentrifugation supernatant (SN) from culture in supplemented LB (Media), denatured SN and neutralized SN (pH 6.0). Bacterial growth was monitored by spectrophotometry (OD 600 nm). Supplemented LB is shown as a control, representing 100% bacterial growth under standard conditions. Bars represent mean value and standard deviation (*n* = 3). Asterisks indicate significant difference compared to the control (*p* < 0.05; one‐way ANOVA followed by Tukey comparisons). (B) Optical density (OD) of *E. coli* (left) and *L. casei* (right) cultures incubated until stationary phase with different dilutions of *L. casei* ultracentrifugation supernatant (SN) and denatured SN obtained from *L. casei* grown in supplemented LB (0.75% glucose, pH 6.0). Bars represent mean value and standard deviation (*n* = 3). The starting pH value is indicated by a line. Asterisks indicate significant difference compared to the control (*p* < 0.05; one‐way ANOVA followed by Tukey comparisons).

To assess the contribution of soluble factors produced by *L. casei* to the antimicrobial activity, we tested the inhibitory effect of *L. casei* ultracentrifugation supernatant (SN) against the growth of *E. coli* and *L. casei*. The SN exhibited a strong inhibitory effect on *E. coli* growth (*p*<0.005) while having no significant impact on *L. casei* growth (*p* > 0.05) (Figure 2A). The antibacterial activity was confirmed to be pH‐dependent, with non‐treated SN (pH 4.0) demonstrating a more potent inhibition than the SN at pH 6 (Figure 2A,B). It is widely recognized that lactobacilli produce lactic acid and other organic acids, which cause a reduction in the pH, thereby inhibiting the proliferation of competing bacteria. Furthermore, lactobacilli are capable of synthesizing various antimicrobial compounds, such as hydrogen peroxide, ethanol, and acetaldehyde, as well as bacteriocins [30]. This study is not the first report to demonstrate the antibacterial activity of lactobacilli supernatant against *E. coli*. The cell‐free supernatant of *L. casei* was previously shown to exhibit antibacterial activity against *E. coli* pathogenic strains [31], and other lactobacilli cell‐free supernatants also showed antimicrobial activity against pathogenic multidrug‐resistant bacteria [32]. However, our work is one of the few studies in the literature that provides explicit evidence regarding the lack of effects of lactobacilli supernatant on the growth of lactobacilli themselves.

The SN adjusted to pH 6, which matches the pH of the media control, still demonstrated an inhibitory effect on *E. coli* growth (Figure 2A). In contrast, this treatment did not impact the growth of *L. casei*. While organic acids likely contribute to the overall antimicrobial effect, this observation indicates that the antibacterial activity is multifactorial, involving both pH‐dependent and pH‐independent mechanisms.

Conversely, the denatured SN had a similar effect on *E. coli* growth to the non‐treated SN, indicating the antibacterial compounds are thermally stable (Figure 2A). Also, a previous study demonstrated that *Limosilactobacillus reuteri* CFS exhibited a greater antimicrobial effect against Gram‐negative bacteria, including *E. coli* ATCC25922, compared to Gram‐positive bacteria [33]. The antimicrobial activity was associated with pH‐dependent, non‐proteinaceous compounds with a molecular weight of less than 3 kDa, while MVs contributed minimally to this effect. These findings align with the present study results for *L. casei* BL23.

It is important to note that the standard medium for culturing *L. casei* is MRS; however, MRS has been shown to have an inherent inhibitory effect on *E. coli* growth due to its acetate content (Figure S2), leading to potential confounding effects in our analysis. To address this issue, we utilized a medium free of acetate—specifically, supplemented LB medium (pH 6, glucose 0.75%)—to collect the *L. casei* SN for all experiments (Figure 2). This approach ensures that the observed antibacterial effects of the SN are attributed exclusively to the bioactive compounds produced by *L. casei*.

It is also worth mentioning that *E. coli* DH5α is a non‐pathogenic, well‐characterized laboratory strain widely used as a baseline model due to its genetic stability and biosafety [34, 35]. According to our results, inhibition of *E. coli* DH5α growth does not automatically translate to effectiveness against pathogenic strains. However, studying these interactions can significantly improve our understanding of bacterial communication and competition within the gut microbiome, leading to new insights into microbiome modulation and probiotics’ mechanisms of action.

### 2.3 *L. casei* MVs are Internalized by *E. coli* and *L. casei*

**FIGURE 3:**
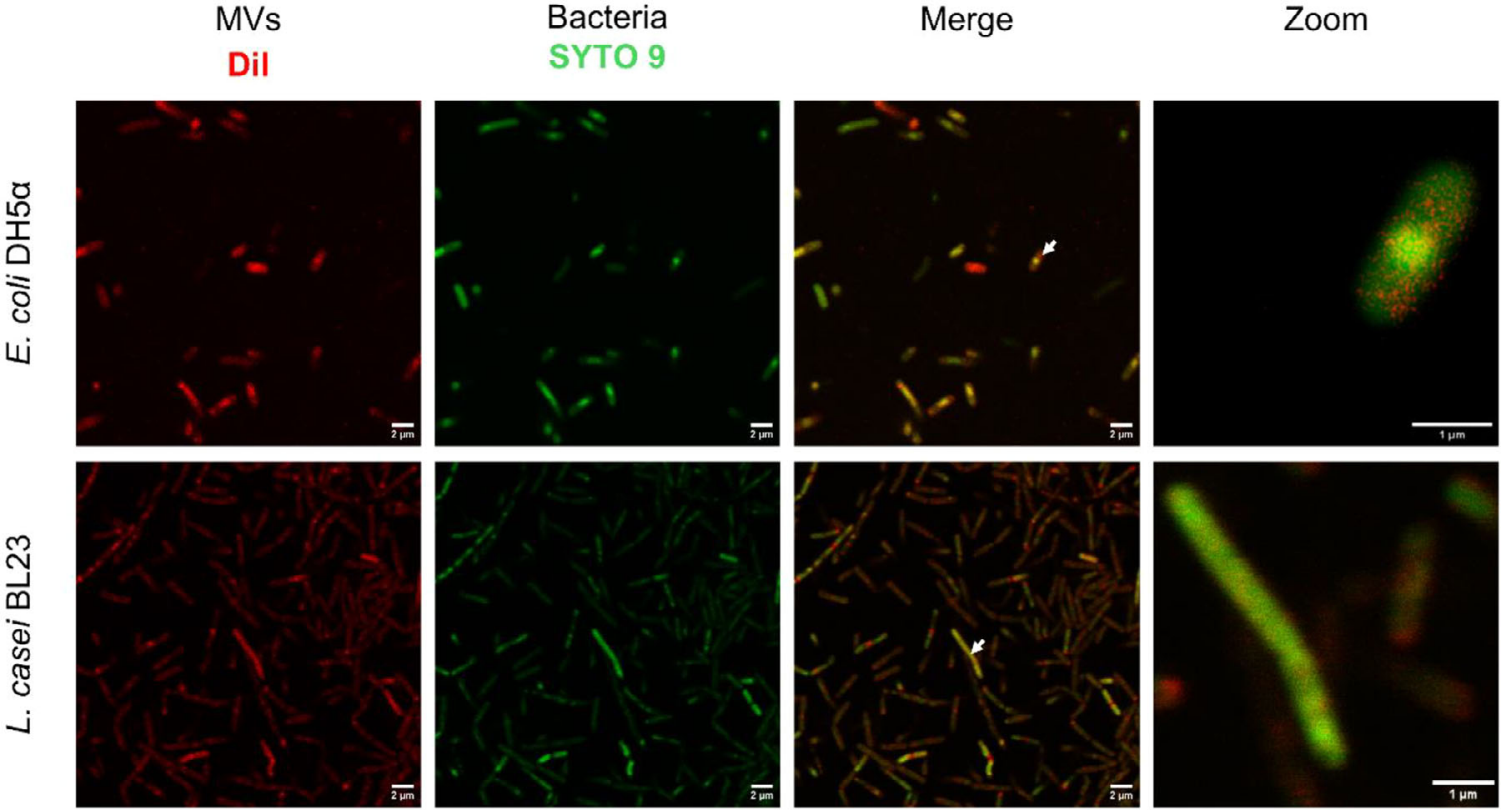
Representative CLSM images of *E. coli* and *L. casei* incubated with *L. casei* MVs. Bacteria were incubated with fluorescently (DiI)‐labeled MVs (10^10^ mL^−1^) for 24 h. Images were captured using consistent laser settings at 561 and 488 nm. Arrows indicate individual bacterial cells, with a corresponding zoomed view presented in the adjacent panel. Scale bars indicate 2 and 1 µm in the zoomed image.

After measuring the antibacterial effect of *L. casei* MVs, we investigated whether these MVs are internalized by both *L. casei* and *E. coli*. Understanding the microbial communication mediated by MVs can reveal how *L. casei *MVs exert their antibacterial effects and modulate the behavior of competing species like* E. coli*. Briefly, *L. casei* DiI‐labeled MVs were incubated with *E. coli* DH5α and *L. casei* BL23 for 24 h. After 24 h incubation, co‐localization of SYTO 9‐labeled bacteria with DiI‐labeled MVs was observed with a Confocal Laser Scanning Microscopy (CLSM) method (Figure 3). The DiI‐labeled MVs were excited using a laser at 561 nm, while SYTO 9‐labeled bacteria were excited at 488 nm. SYTO 9 was employed to visualize both live and dead bacteria during microscopy. Control samples, where bacteria were incubated with PBS (without MVs), did not exhibit co‐localized fluorescence (Figure S3). Overall, these results indicate that *L. casei* MVs were internalized by *E. coli* and *L. casei*, as confirmed by Z‐stack imaging observations (Figure S4).

We further studied the early‐stage interaction of *L. casei* MVs with *E. coli* after a 30 min incubation, before bacterial replication could occur (Figure S5). In this setup, DiI‐labeled MVs were incubated with DiO‐labeled *E. coli* for 30 min at 37°C. CLSM images revealed co‐localization of MVs with some *E. coli* cells, as indicated by arrows in the images. The percentage of bacteria showing co‐localization at this early time point was notably lower compared to the 24‐h incubation period, indicating that the extent of MV‐bacteria interactions progressed over time. In contrast to MV uptake by mammalian cells, MV uptake by bacterial cells has not been thoroughly investigated. However, it is well‐known that MVs play a significant role in interbacterial communication, particularly in processes such as horizontal gene transfer and the exchange of signaling molecules [36, 37, 38]. In this report, uptake analyses indicate that *L. casei* MVs are internalized by *E. coli* in a manner comparable to their internalization by *L. casei*; the molecular mechanisms underlying the internalization of MVs into bacterial cells still remain to be elucidated, but emerging evidence suggests that direct membrane fusion is one of the mechanisms involved [39, 40]. Notably, our results suggest MVs from Gram‐positive bacteria, such as *L. casei*, can also be internalized by Gram‐negative bacteria like *E. coli*. These findings contribute to a growing body of evidence supporting the role of bacterial MVs in mediating interspecies interactions. Nevertheless, we cannot assert that uptake is a prerequisite for the antibacterial effects observed. Alternative mechanisms independent of internalization may contribute, necessitating further functional validation to delineate these relationships.

### 2.4 MVs did not Result in Cytotoxicity or Decreased Cell Viability in Peripheral Blood Mononuclear Cells

While *L. casei* BL23 is generally recognized as safe as probiotic strains of lactobacilli, the safety of its MVs requires separate evaluation. To evaluate the potential cytotoxicity of *L. casei* MVs on Peripheral Blood Mononuclear Cells (PBMCs) we conducted two key assays: lactate dehydrogenase (LDH) and PrestoBlue assays, using Triton X 1% as a positive control and phenol red‐free RPMI 1640 medium as a negative control.

**FIGURE 4:**
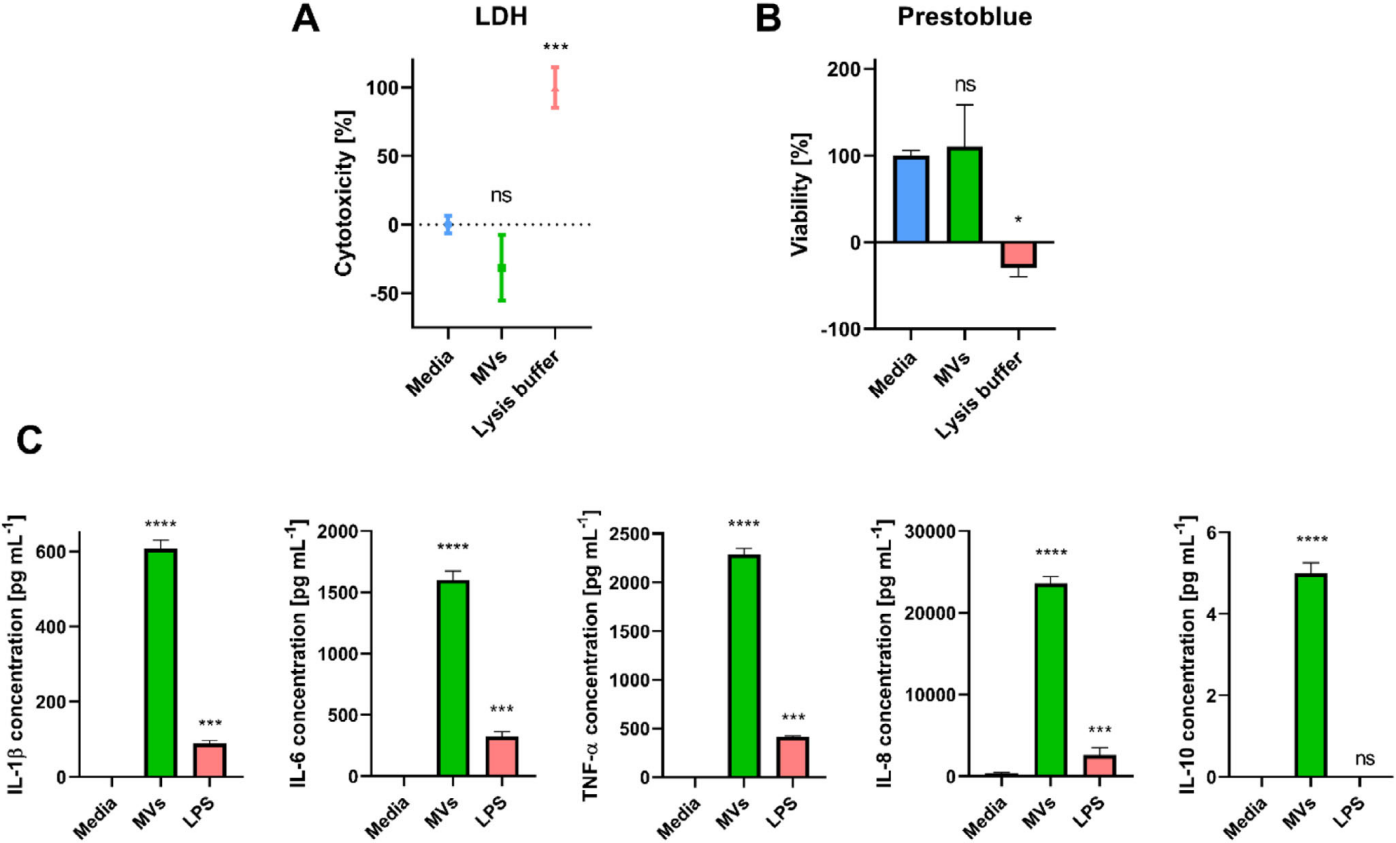
(A) Cytotoxicity [%] of *L. casei* MVs on PBMCs after 24 h of incubation, assessed by LDH assay. Mean ± SD, One‐way ANOVA followed by Tukey *post‐hoc* test (*n* = 3). Asterisks indicate significant difference compared to the control (media) (*p *< 0.05). (B) Cell viability of PBMCs after 24 h of incubation with *L. casei* MVs, assessed by PrestoBlue assay. Mean ± SD One‐way ANOVA followed by Tukey *post‐hoc* test (*n* = 3). Asterisks indicate significant difference compared to the control (media) (*p *< 0.05). (C) Cytokine production in MV‐treated PBMCs was measured by flow cytometry. Data are presented as estimated marginal means with standard error bars. Bars represent cytokine concentration [pg mL^−1^] for each treatment condition. Mean ± SD, One‐way ANOVA followed by Tukey *post‐hoc* test (*n* = 3). Asterisks indicate significant difference compared to the control (media) (*p *< 0.05).

In the LDH assay, despite the positive control, treatment with MVs for 24 h did not result in an increase in LDH release compared to the negative control, confirming the biocompatibility of *L. casei* MVs (Figure 4A).

To further assess cell viability, we utilized the PrestoBlue assay, a method that quantifies metabolic activity in cells. Absorbance readings at 565 nm indicated that PBMCs treated with MVs maintained viability comparable to that of the medium control, whereas 1% Triton X resulted in a marked decrease in cell viability (Figure 4B).

The results from both assays support the conclusion that MV treatment neither induces cytotoxicity nor decreases cell viability in PBMCs, indicating *L. casei* MVs’ potential for further therapeutic applications.

### 2.5 MVs Elicit a Predominant Pro‐Inflammatory Immune Response with Concurrent IL‐10 Production in PBMCs

MVs, as naturally occurring nanoparticles, contain a variety of immunostimulatory ligands, known as microbial‐associated molecular patterns, which can activate the host's immune system [4]. To assess the immune response elicited by *L. casei* MVs, we treated PBMCs with MVs and measured cytokine release using the Cytometric Bead Array. Our results revealed that treatment with MVs significantly elevated the concentrations of both pro‐inflammatory cytokines, namely TNF‐α, IL‐6, IL‐8, and IL‐1β, as well as the anti‐inflammatory cytokine IL‐10, compared to a media control (Figure 4C). Interestingly, we also observed that the levels of pro‐inflammatory cytokines in the MV treatment group surpassed those found in a lipopolysaccharide (LPS) control group. This finding suggests that MVs may activate immune pathways more effectively than LPS alone, which is traditionally recognized for its potent immunogenic properties.  The presence of IL‐10 may represent a regulatory mechanism attempting to modulate the inflammatory response.

To further elucidate the effects of MV treatment on cytokine transcription rates, we measured IL‐1β and IL‐6 mRNA levels in treated PBMCs using quantitative real‐time PCR (qPCR). As shown in Figure S6, MV treatment significantly increased IL‐1β and IL‐6 expression compared to the media control, in accordance with the elevated protein levels of these cytokines detected in the supernatants of treated PBMCs (Figure S6), indicating that the increased mRNA levels correlate with higher protein production. This suggests that MV treatment effectively enhances both transcriptional and translational expression of these pro‐inflammatory cytokines.

These findings provide a comprehensive picture of the immune response triggered by *L. casei* MVs on PBMCs, from cytokine mRNA expression level to cytokine secretion. MVs induced the release of both pro‐inflammatory and anti‐inflammatory cytokines in PBMCs. The dual immunomodulatory action of *L. casei* MVs may be due to their complex structure, which includes surface lipoteichoic acid (LTA) and peptidoglycan among other immunogenic components [5, 16]. Remarkably, LTA, a component found in *L. casei* BL23 MVs, can activate Toll‐like receptor 2 (TLR2), leading to the stimulation of pro‐inflammatory pathways as well as regulatory mechanisms that enhance anti‐inflammatory responses [41, 42]. While most studies in literature focused on the anti‐inflammatory effects of probiotic MVs, the release of pro‐inflammatory cytokines such as IL‐1β, TNF‐α, and IL‐6 have been found beneficial in certain contexts, such as enhancing the immune system's ability to fight infections or cancer [43]. Notably, *L. casei* MVs are rich in the protein p75 [14, 16], a peptidoglycan hydrolase shown to be essential for efficient phagocytosis of *L. casei* by human dendritic cells and for inducing secretion of pro‐inflammatory cytokines (TNF‐α, IL‐1β, IL‐6) as well as T cell‐polarizing cytokines (IL‐10, IL‐12, IL‐23), as demonstrated by Tóth et al. [41], highlighting its critical function in shaping immune responses pivotal for host defense [44]. Bäuerl et al. [20] have reported that *L. casei* MVs at high doses induce a moderate increase in NF‐κB expression in intestinal epithelial cells (HT‐29). It is reasonable to infer that PBMCs, being more sensitive to immunomodulatory stimuli, may exhibit a more robust response to MV stimulation.

Importantly, the immunostimulatory effects of *L. casei* MVs may further contribute to combating infections by promoting immune cell activation and pathogen clearance; for example, pro‐inflammatory cytokines enhance the microbicidal activity of macrophages [45, 46, 47]. Indeed, the use of immunostimulatory antibacterial biomaterials to enhance bactericidal activity represents a promising strategy to synergistically accelerate pathogen clearance during the acute and chronic phases of infection [48].

Together, our results are in accordance with previous studies on probiotic‐derived MVs [43, 49, 50] and implies the potential of *L. casei* BL23 MVs for infection treatment, vaccine development or as immunotherapy adjuvants [43].

### 2.6 *L. casei* MVs Purified by Size Exclusion Chromatography Contain Lipoteichoic Acid

To confirm the presence of the Lipoteichoic acid (LTA) in *L. casei* MVs, the LTA level of MVs purified with SEC, UC pellet, and the parent bacteria all concentrated to the same protein concentration were measured via a dot blot assay. Surprisingly, LTA was detected only in the purified MVs (Figure S7), which can justify the reported immunomodulatory effects of lactobacilli MVs through the interaction of LTA with the immune receptor TLR2. Notably, LTA has also been identified as a component of other lactobacilli MVs and is associated with their immunomodulatory effects on various cell types [16, 41, 49]. 8We assume that the reason behind detecting no LTA in the UC pellet and the bacteria was the relatively high protein levels in these samples compared to a very low amount of LTA. Accordingly, we suggest that LTA can serve as an index for purity of *L. casei* MVs and has the potential to be considered in quality control method development for these postbiotics.

### 2.7 MVs are Internalized by Macrophages in a Slow Time‐Dependent Manner

**FIGURE 5:**
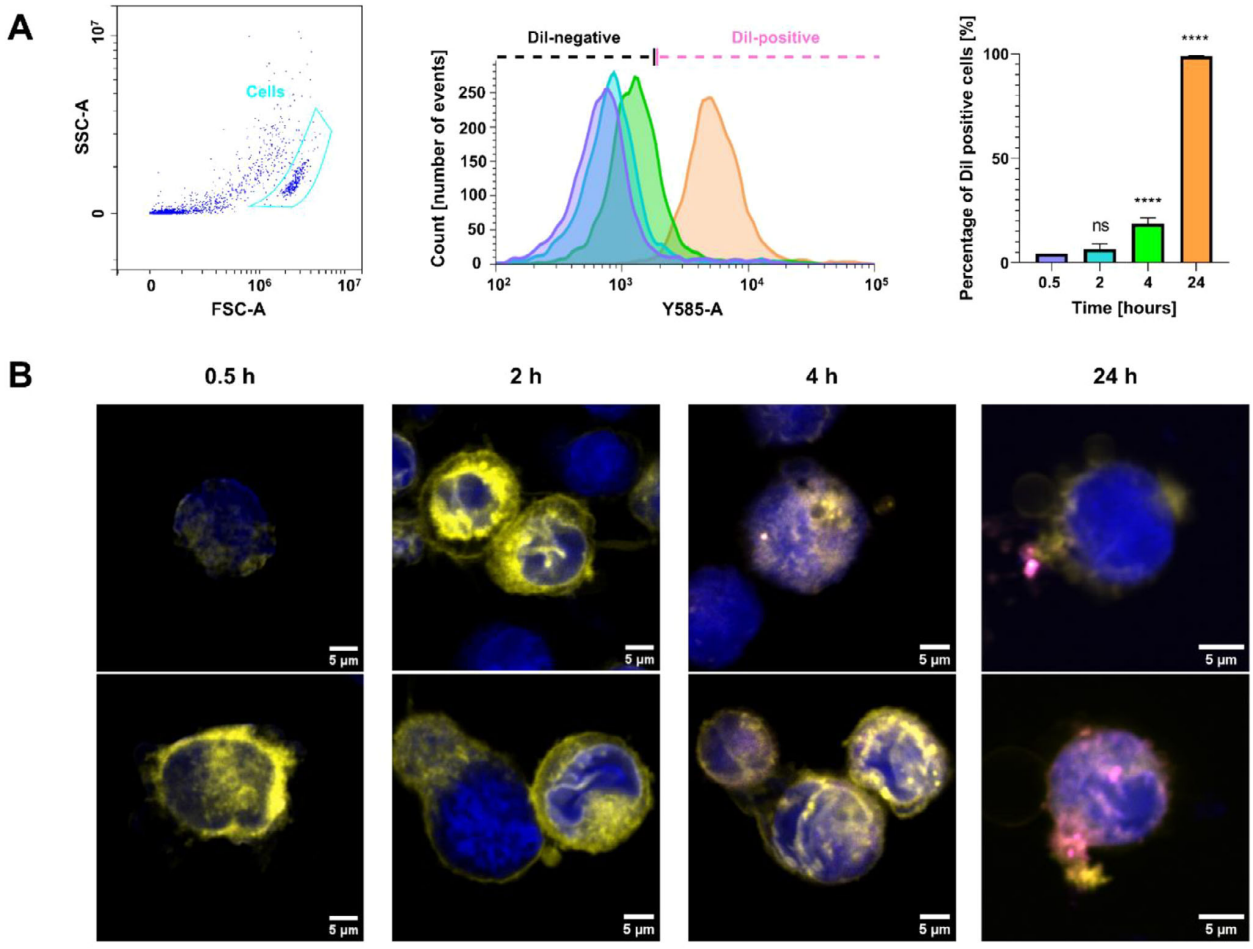
(A) Quantification of MVs’ uptake by macrophages, utilizing flow cytometry after 0.5, 2, 4, and 24 h of incubation. The left panel displays the gating strategy based on forward and side scatter (FSC vs. SSC), with the gated region highlighting the cells. The middle panel histograms represent the uptake of MVs by macrophages at different time points, according to the fluorescence intensity of DiI‐positive macrophages. The bar plot in the right panel features a comparative overview of DiI‐positive macrophages (mean ± SD; *n* = 3). Asterisks indicate a significant difference compared to the percentage of DiI‐positive cells at 0.5 h (Repeated measures ANOVA, *p *< 0.05). (B) Visualizing the uptake of MVs by macrophages with CLSM, after 0.5, 2, 4, and 24 h of incubation. The representative images show the progressive uptake of MVs by macrophages at different time points. The macrophage cell membrane is stained in yellow with DiD, the nuclei are stained in blue with DAPI, while DiI‐labeled MVs are displayed in magenta.

Understanding the interactions between *L. casei* MVs and macrophages is crucial, as these interactions can significantly influence the immune response and therapeutic efficacy of MVs. To better understand the mechanisms and dynamics of how *L. casei* MVs interact with macrophages, we monitored the uptake of MVs by THP‐1 monocytes derived‐macrophages for 24 h. DiI‐labeled MVs were incubated with macrophages for 0.5, 2, 4, and 24 h at 37°C. After 24 h incubation, approximately 98% of the macrophages were DiI‐positive according to the flow cytometry results, indicating that nearly all macrophages internalized MVs (Figure 5A). The internalization of MVs by macrophages occur in a time‐dependent manner. Also, CLSM images showed that *L. casei* MVs were internalized by macrophages to a similar extent (Figure 5B). MVs appeared to be attached to the membrane, in the cytoplasm and inside the nuclei, according to Z‐stack confocal imaging (Video S1).

Despite significant research on the interactions between MVs and macrophages, less attention has been given to the internalization rates of lactobacilli MVs. Until now, only few studies have demonstrated the internalization of lactobacilli MVs by macrophages [51, 52]. In our work, the rate of internalization of *L. casei* MVs after 2 h (5%) is considered slow compared to typical rates observed for bacterial MVs in similar studies. For instance, one study reports that 40% of THP‐1 derived macrophages internalized *Staphylococcus aureus* MVs after less than 2 h incubation [53]. A reduced uptake of *L. casei* MVs by macrophages of the reticuloendothelial system could potentially prolong their circulation time [54]. However, given that *L. casei* MVs induced the release of pro‐inflammatory cytokines in PBMCs, prolonged circulation may increase the risk of heightened immune activation or hyperinflammatory responses [55]. Therefore, while decreased clearance might enhance their utility as therapeutic agents or delivery vehicles, balancing immune stimulation and safety will be critical in the future for their effective application [56]. Further studies are needed to confirm the exact uptake mechanisms of *L. casei* MVs in macrophages.

### 2.8 MVs Preserve the Overall Epithelial Barrier Integrity

**FIGURE 6:**
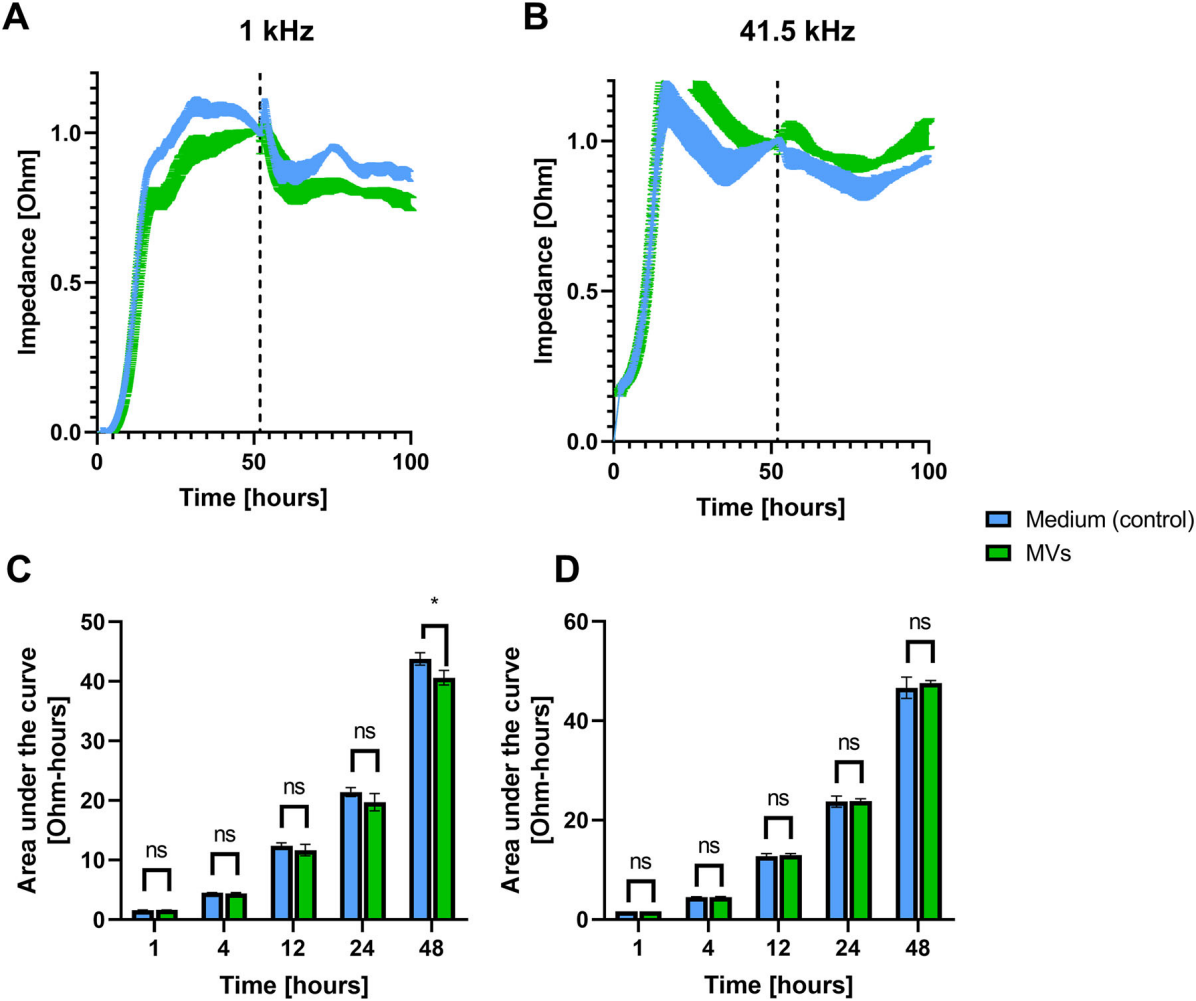
Effect of *L. casei* MVs (1 × 10^10^) compared to medium on electrical impedance of intestinal epithelium. (A) Changes in normalized impedance [Ohm] over time measured at a frequency of 1 kHz. The dashed line represents the starting incubation time. Values are shown as mean (± SD) from four replicates. (B) Changes in normalized impedance [Ohm] over time measured at a frequency of 41.5 kHz. The dashed line represents the starting incubation time. Values are shown as mean (± SD) from four replicates. (C) Cumulative change in normalized impedance values (Area under the curve [Ohm‐hours]) at 1 kHz at different time points. *Significant difference compared to the control (Two‐way Repeated Measures ANOVA, *p* = 0.0419, *n* = 4). (D) Cumulative change in normalized impedance values (Area under the curve [Ohm‐hours]) at 41.5 kHz at different time points (Two‐way Repeated Measures ANOVA, *p *> 0.05, *n* = 4).

Maintaining intestinal barrier integrity is essential for preventing serious conditions like infections and inflammations [57]. It has been reported that consuming probiotics leads to a balanced microbiome and contributes to maintaining this epithelial barrier integrity [58]. To investigate if the MVs are involved in this process, we studied the impact of *L. casei* MVs on the impedance in Caco‐2 cell monolayers. The Axion Maestro Z system enabled continuous monitoring of cells in a controlled environmental chamber, facilitating impedance measurements over time in a 96‐well plate format [59]. As represented by impedance measurements at 41.5 kHz, MVs did not affect the cell coverage and overall integrity of the epithelial barrier after 48 h incubation with a Caco‐2 monolayer (Figure 6). In contrast, a slight decrease by 5% in impedance was observed at 1 kHz after 48 h. This slight decrease might imply minor disruptions in tight junction functions. This observation can be attributed to alterations in the expression of tight junction proteins, which does not necessarily indicate a detrimental effect on the integrity of the intestinal barrier. For example, OMVs from the probiotic *E. coli* Nissle 1917 upregulated ZO‐1 and claudin‐14 while downregulating claudin‐2, which may reflect a nuanced modulation of tight junction integrity, despite providing protection against barrier disruption induced by enteropathogenic *E. coli* [60].

Overall, our results are consistent with the effects observed from other studies on probiotic MVs [7]. However, to our knowledge, no other research has specifically investigated the impact of probiotic MVs on impedance over a duration of up to 48 h and at two different frequencies. Nevertheless, several studies demonstrated how probiotic MVs were effective in restoring the barrier integrity in models where the epithelial barrier integrity was compromised by pathogens [49, 60].

### 2.9 MV Treatment Prolonged the Lifespan of *C. elegans* Challenged with *Pseudomonas aeruginosa*

*C. elegans* is a simple, well‐characterized nematode model widely used to study host‐pathogen interactions due to its conserved immune pathways [61]. Its use involves minimal ethical concerns, making it ideal for investigating the effects of probiotic MVs on survival during exposure to pathogen toxins [61, 62]. To evaluate the role of *L. casei* MVs in protecting *C. elegans* against death caused by bacterial toxins from *P. aeruginosa*, we performed a fast‐killing assay [61]. This assay is conducted on Peptone Glucose Sorbitol (PGS) agar, a high‐osmolarity medium that facilitates the rapid release of *P. aeruginosa* toxins, leading to the quick death of the worms within several hours. *E. coli* OP50 (standard food source for *C. elegans*) was used as a negative control.

**FIGURE 7:**
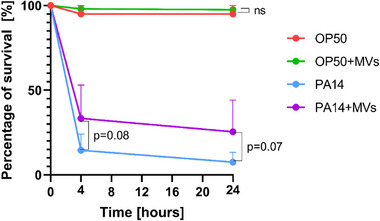
Effect of *L. casei* MVs on the survival of *C. elegans* infected with *P. aeruginosa* PA14. *E. coli* OP50 served as a nonpathogenic control. Data are represented as a mean + SD. The level of significance for each timepoint (4 and 24 h) and bacteria (*P. aeruginosa* PA14 or *E. coli* OP50) was analyzed by Student T‐test. P‐values of the single comparison between PA14 and PA14+MVs for each time point are indicated in the graph.

As shown in Figure 7, approximately 93% of nematodes treated only with *P. aeruginosa* PA14 died within 24 h, while 95% of the wild type animals fed with *E. coli* OP50 remained alive (Figure 7). Moreover, the chart shows that the lifespan of animals treated with OP50 and *L. casei* MVs‐was similar to that of animals being treated only with OP50, indicating that *L. casei* MVs are non‐toxic for *C. elegans*.

Notably, when nematodes were co‐exposed to *P. aeruginosa* and *L. casei* MVs, nearly 25% of the animals survived. Statistical analysis reveals a discernible trend in these survival outcomes, reaching a near‐significant protective effect (p = 0.07) upon 24 h of bacterial incubation. Our observation suggests a potential biological effect of *L. casei* MVs in extending the survival of *C. elegans* when exposed to *P. aeruginosa* PA14. Further studies in mammalian models are necessary in the future to fully understand immune cell‐mediated mechanisms and the translational relevance of *L. casei* MVs in higher organisms.

The role that lactobacilli MVs play in the extension of lifespan in worms has been documented in several studies. A study demonstrated that MVs from *L. acidophilus* and *L. plantarum* could prolong the survival of *C. elegans* under exposure to vancomycin‐resistant *Enterococcus faecium*. The treatment with these MVs led to increased transcription of host defense genes, specifically *cpr‐1* and *clec‐60*, which play protective roles against bacterial infections. This supports the idea that lactobacilli MVs can modulate the immune response and improve the overall health of the nematodes, contributing to lifespan extension [63].

To our knowledge, the effects of lactobacilli‐derived MVs particularly on reducing the toxic mechanisms of pathogenic bacteria have not yet been studied. However, lactobacilli have been shown to protect *C. elegans* by inhibiting the expression of enterotoxin genes in enterotoxigenic *E. coli*, which correlated with increased survival rates of the nematodes exposed to the pathogenic bacteria [64]. Prior studies showed *L. casei* supernatant inhibits toxin production and quorum sensing in the less virulent *P. aeruginosa* PAO1 strain, supporting its protective role [65]; the high virulence of the strain used in our study, PA14, likely accounts for the observed trend. These results suggest *L. casei* MVs may enhance nematode survival by mitigating bacterial pathogenicity.

## 3 Conclusion

Our investigation significantly enhances the understanding of *L. casei* MVs as a promising biocompatible avenue for antibacterial treatment and immune stimulation. Purified *L. casei* MVs and supernatant demonstrated selective antibacterial activity against *E. coli*, warranting further exploration of the underlying molecular mechanisms. In future research, it would be critical to investigate potential synergies with other antimicrobial agents to maximize their effectiveness, as well as the antibacterial effect against *E. coli* pathogenic strains. In our work, we found a significant antimicrobial activity of *L. casei* MVs but it is crucial to acknowledge that real‐world applications may encounter additional challenges, such as antibiotic‐resistant and biofilm‐forming bacteria. Moreover, *L. casei* MVs elicited a robust immune response in human PBMCs, indicating their promising potential as an antibacterial therapeutic that could exert dual effects by additionally enhancing the immune defense against pathogens. In this study, the observation of an extended lifespan in *C. elegans* treated with *L. casei* MV provides valuable insights into their potential health benefits in a living organism. This research lays a solid foundation for future investigations aimed at fully elucidating and harnessing the therapeutic and prophylactic potential of these bacterial MVs in clinical applications.

## 4 Experimental Section

### 4.1 *L. casei* BL23 Culture and MV Isolation

*Lacticaseibacillus casei* BL23 was isolated in Man‐Rogosa‐Sharpe (MRS; BD Difco, Sparks, MD, USA) agar plates. Bacterial suspensions (initial OD = 0.15; 260 mL) were cultured for 48 h at 37°C to reach the stationary phase of growth. The culture was centrifuged at 4500 g for 20 min at 4°C. The supernatant was filtered in sterile conditions through a Stericup Quick Release 0.45 µm system (Merck, Germany) to remove cell debris and exopolysaccharides. The filtered supernatant was spun at 110 000 g for 2 h at 4°C (Type 45 Ti Fixed‐Angle Rotor, Optima XPN‐80 Ultracentrifuge, Beckmann Coulter, Germany). The pellets were washed with sterile PBS and subjected to a second ultracentrifugation step to eliminate any residual media contaminants. Finally, pellets were resuspended in sterile PBS to obtain the final UC pellet. For further studies, the ultracentrifugation supernatant (SN) was collected and stored at 4°C [14].

MVs were further purified from soluble proteins by SEC using a 10 mL Sepharose CL‐2B (GE Life science UK) column [66]. Twenty eluted fractions of 750 µL were collected in 1.5 mL tubes. To increase particle concentration, SEC fractions containing MVs were further pelleted by ultracentrifugation at 110 000 g for 2 h at 4°C (Optima MAX‐XP Ultracentrifuge) and MVs were resuspended in sterile PBS.

### 4.2 Protein Concentration Measurement

To confirm the effectiveness of MV purification by SEC and determine the protein concentration in each eluted fraction, the ROTIⓇQuant Universal Kit (Carl Roth, Germany) was used, which is based on a similar principle to standard Bicinchoninic acid assay. Protein concentration was determined in triplicate by comparing it to a bovine serum albumin standard curve with concentrations of 5, 25, 50, 125, 250, 500, 750, and 1000 µg mL^−1^.

### 4.3 Transmission Electron Microscopy (TEM)

TEM was performed on unpurified MVs and on MV samples purified by SEC. Negative stain room temperature TEM was performed according to Arnold et al. [67]. Briefly, 3 µL of sample was added to a continuous carbon‐coated 300 mesh copper grid (Science Service, Munich, Germany) treated with plasma for 15s at 25 mV for negative glow discharge. Excess liquid was immediately blotted off with filter paper and the grid was washed twice with 5 µL of an aqueous 2% uranyl acetate solution (Merck Millipore, Billerica, MA, USA). After air drying, samples were inserted into a JEOL 1400 Plus transmission electron microscope (JEOL Germany, Munich, Germany) operating at 120 kV and images were acquired at a nominal magnification of 30 000‐fold.

### 4.4 Nanoparticle Tracking Analysis (NTA)

Particle size and concentration were measured using a Zetaview PMX‐220 NTA instrument (Particle Metrix, Germany) and analyzed using the Zetaview (version 8.05.16 SP3) software. The instrument was calibrated by using a known concentration of polystyrene 100 nm bead suspension. The SEC‐eluted fractions containing MVs were diluted with PBS for analyses. Particle size and concentration were measured at 11 frames per cycle under a sensitivity of 80 and a shutter value of 100 in all samples to guarantee the results were comparable.

### 4.5 Storage Stability of MVs

MV samples were stored in PBS at 4°C for 4 months to evaluate their stability. Initially, protein concentration was quantified using BCA, and particle size and concentration were assessed via NTA, establishing baseline values corresponding to 100%. Following the 4‐month storage period at 4°C, the same measurements were repeated to determine any changes.

### 4.6 Antimicrobial Assays

The antimicrobial effect of *L. casei* BL23 MVs was analyzed against *Escherichia coli* DH5ɑ and *L. casei* BL23. To this end, bacterial growth was monitored through the measurement of optical density at 600 nm according to Schulz et al. (2018) with some modifications [34].

Briefly, 100 µL of a bacterial suspension in broth (Lysogeny Broth (LB) for *E. coli* DH5ɑ and MRS for *L. casei* BL23) with 1 × 10^7^ CFU mL^−1^ was incubated with (1) 100 µL UC pellet (2,5 × 10^12^ particles mL^−1^), (2) 100 µL MVs (5 × 10^11^ particles mL^−1^), (3) 100 µL ultracentrifugation supernatant (SN) (4) 100 µL denatured ultracentrifugation supernatant (Denatured SN), 5) 100 µL neutralized ultracentrifugation supernatant (Neutralized SN), in a Greiner 96 Flat Bottom Transparent Polystyrene well plate. Sterile PBS (pH 7.4) was applied as a negative control for the UC pellet and MVs treatment, and the culture medium was applied as a negative control for the SN treatment. The unused wells of the microplate were filled with sterile PBS to prevent evaporation. The plate was incubated overnight at 37°C while shaking in a Tecan Infinite 200 Pro Plate reader, and absorbance at 600 nm was measured every 30 min until the beginning of the stationary phase: after 15 h for *E. coli* and after 18–20 h for *L. casei*. Additionally, plate counts were performed using LB and MRS agar plates after 20 h of incubation (stationary phase) to assess the viable bacterial counts of bacteria treated with UC pellet, MVs and PBS (control).

Since MRS is a selective growth medium for lactobacilli and inhibits *E. coli* growth by itself, we also tested the antimicrobial activity of the SN from *L. casei* grown in a supplemented LB medium. To achieve this, the same procedures used for the isolation of *L. casei* MVs in MRS media were followed. The supplemented LB media was prepared by adding 0.75% glucose to standard LB media and adjusting the pH to 6.0 using 1 m hydrochloric acid (HCl) to enhance the growth of *L. casei*. The antimicrobial activity of the SN and denatured SN against *E. coli* and *L. casei* was tested at different dilutions in PBS.

### 4.7 Thermal Treatment and pH Adjustment of the Ultracentrifugation Supernatant

To evaluate the effect of pH on the antibacterial activity of the ultracentrifugation supernatant (SN), the pH of the SN was measured and adjusted to 6.0 with 1 m NaOH (same pH as the media control). To assess the thermal stability of the antibacterial compounds in the supernatant, aliquots were subjected to heat treatment at 121°C for 15 min, resulting in a denatured supernatant

### 4.8 Confocal Laser Scanning Microscopy

*L. casei* MVs were labeled and their interaction with *E. coli* DH5α and *L. casei* BL23 was assessed according to Schulz et al. [35]. Briefly, the UC pellet (1 mL) was incubated with 2 µL DiI (Vybrant DiI Cell‐labelling solution) for 30 min at 37°C and the free dye was removed by SEC. The fluorescence intensity of each fraction was measured using a Tecan Infinite 200 Pro Plate reader (Ex/Em: 490/570 nm, gain 177, 25 flashes, 20 µs integration, Z‐position: 20 000 µm) and the most concentrated MV fraction was used for further analyses. After incubating *E. coli* and *L. casei* with DiI stained MVs for 24 h at 37°C, bacteria were labeled with 1.5 µL SYTO 9 Green fluorescent nucleic acid stain and incubated at 37°C for 10 min. After centrifugation at 9500 g for 5 min, bacteria were fixed with 4% paraformaldehyde for 10–15 min at 37°C. After three washes with PBS, 10 µL of the sample was applied on a coverslip with a drop of BacLight mounting oil (Thermo Fisher Scientific, MA). Images were taken using a confocal laser scanning microscope (FV3000, Olympus, Japan) with 20× (UPlanXApo 20x/0.8, Olympus, Japan) and 100× (UPlanXApo 100x/1.45 Oil, Olympus, Japan) lenses. A laser with an excitation wavelength of 561 nm (filter: 570–670 nm) was set up to visualize DiI‐stained MVs and a laser with an excitation wavelength of 488 nm (filter: 500–540 nm) was used to visualize SYTO 9 stained bacteria. Z‐stack images were analyzed using Imaris 9.9.0 software (Oxford Instruments, UK), which enabled advanced visualization and processing capabilities.

To study the early stage of the interaction with *L. casei* MVs, *E. coli* (2 × 10^8^ CFU mL^−1^) was stained with DiO and co‐incubated with DiI‐stained *L. casei* MVs for 30 min at 37°C. After centrifugation at 9500 g for 5 min, the pellet was resuspended in PBS and applied on a coverslip with a drop of BacLight mounting oil (Thermo Fisher Scientific, MA). Images were taken using a confocal laser scanning microscope (FV3000, Olympus, Japan) with 20× (UPlanXApo 20x/0.8, Olympus, Japan) and 100× (UPlanXApo 100x/1.45 Oil, Olympus, Japan) objectives. A laser with an excitation wavelength of 561 nm (filter: 570–670 nm) was set up to visualize DiI‐stained MVs and a laser with an excitation wavelength of 488 nm (filter: 489–545 nm) was used to visualize DiO‐stained bacteria. ImageJ v1.53 was utilized to further process and analyze the acquired images.

### 4.9 Isolation of Peripheral Blood Mononuclear Cells from Buffy Coats

Peripheral Blood Mononuclear Cells (PBMCs) were isolated from healthy adult blood donors at the Blood Donation Center in Erlangen, Germany. The use and handling of human materials were approved by the local Ethics Committee (approval no. 357_19 B; State Medical Board of Registration, Erlangen, Germany). PBMC isolation was performed using density gradient centrifugation [49]. Briefly, blood was diluted with an equal volume of‐ RPMI media (without phenol red, fetal bovine serum and antibiotics). The diluted blood was distributed in two 50‐mL Leucosep tubes (Greiner Bio‐One, Germany), preconditioned with 15 mL of the lymphocyte separation medium 1077 (PromoCell, Germany). The tubes were centrifuged at 300 g for 30 min at room temperature with the brake off. The PBMC layer was carefully aspirated and washed twice with RPMI, single cells were obtained by filtering through a 40 µm‐cell strainer (Starlab, Germany), and concentrated by centrifugation at 250 g for 10 min. The final PBMC pellet was resuspended in 2 mL RPMI.

### 4.10 Assessment of PBMC Viability

The count and viability of PBMCs were assessed using the Trypan Blue exclusion assay. 10 µl of the 100‐fold diluted PBMC suspension, was mixed with an equal volume of 0.4% Trypan Blue solution (Carl Roth, Germany) and incubated at room temperature for 5 min. Then, 10 µL of the samples were loaded onto a Neubauer hemocytometer for counting. Viable cells remained unstained, while non‐viable cells took up the dye and appeared blue. The total number of viable and non‐viable cells was counted across multiple squares, and cell viability was calculated using the formula:

$$\begin{array}{ccc} & & \mathrm{Viability}\left(\%\right)=\\ & & \left(\mathrm{Number}\;\mathrm{of}\;\mathrm{viable}\;\mathrm{cells}/\mathrm{Total}\;\mathrm{number}\;\mathrm{of}\;\mathrm{cells}\right)\times 100 \end{array}$$

This method provided a reliable assessment of PBMC viability, essential for subsequent experimental analyses.

### 4.11 Cytotoxicity and Viability Assays on PBMCs after Treatment with MVs

To assess the viability and cytotoxic effects of MVs on PBMCs, 10^5^ cells were treated with MVs (1×10^11^ particles) for 24 h.

After the treatment period, cytotoxicity assay was performed using the lactate dehydrogenase (LDH) assay on the culture supernatant according to Cytotoxicity Detection Kit^PLUS^ instructions. Phenol red free RPMI 1640 medium (ThermoFisher) was used as a negative control and Triton X 1% (w/v) (lysis buffer), as a positive control.

On the other hand, a viability detection assay was performed using the PrestoBlue assay. 90 µL of the cell suspension was incubated with 10 µL of PrestoBlue Cell Viability Reagent (ThermoFisher) at 37°C for 10 min. After incubation, the absorbance at 565 nm was measured, and the viability was determined relative to the media control and Triton X 1% (w/v) (negative control).

### 4.12 Cytokine Release and Expression Analyses after Treatment with MVs

For cytokine analyses, 10^6^ PBMCs were treated with either MVs (1 × 10^11^ particles), LPS (1 µg mL^−1^) as positive control, or only the media as negative control for 4 h at 37°C. Supernatants and cells from each well were separately collected and stored at ‐80°C, for further analysis of cytokine release at the protein level and gene expression, respectively. Cytokine concentrations in the supernatants were measured using the CBA assay (BD Cytometric Bead Array, Human Inflammatory Cytokines Kit) in a flow cytometer (Cytoflex SRT, Beckmann Coulter, USA). The experimental procedures were conducted according to the manufacturer's instructions.

The sedimented PBMCs were utilized to measure the cytokines’ mRNA levels. Total RNA was extracted using the Monarch Total RNA Miniprep Kit (New England BioLabs, USA). Following the measurement of the RNA content in each sample using a NanoDropTM 2000 spectrophotometer, 300 ng RNA was used to synthesize cDNA using the RevertAid First Strand cDNA Synthesis Kit and hexamer primer (ThermoScientific, USA), following the manufacturer´s instructions. Reverse transcription was carried out for 1 h at 42°C in a FlexCycler 2 PCR thermocycler (Analytic Jena GmbH). The generated cDNA samples were stored at −20°C. Quantitative real‐time PCR (qPCR) was performed using FAST SYBR Green Mastermix Kit (Applied Biosystems, Germany) to assess the expression levels of the human inflammatory cytokines IL‐1β and IL‐6 using an AriaMx Real‐Time PCR device (Agilent Technologies, USA). The sequences for the forward and reverse primers for human reference 18S ribosomal RNA (18S rRNA) and each cytokine are listed in Table S1 (Eurofins, Luxembourg). For each sample, qPCR was performed using a starting amount of 50 ng cDNA and the following reaction cycles: 95°C for 3 min, then 40 amplification cycles (95°C for 5 s followed by 60°C for 40 s), and finally 1 melting cycle (95°C for 30 s followed by 60°C for 30 s and finally 95°C for 30 s). Fluorescence was quantified during each amplification. AriaMx Software (Agilent Technologies, USA) was used to determine the threshold cycle (Ct) value for each treatment. For subsequent analysis and to establish a basis for statistical tests, we utilized the relative quantification (RQ) value using the 2^−ΔΔCt^ method [68] and the housekeeping gene 18S rRNA as the reference gene for normalization (Equation 1).

ΔCt=Ctgene−Ctreferencegene

ΔΔCt=ΔCtsample−ΔCtcontrol

(1) $$\mathrm{RQ}=2^{-\Delta \Delta \mathrm{Ct}}$$

Equation (1) shows Relative quantification (RQ) value calculation.

### 4.13 THP‐1 Cell Culture and Differentiation

THP‐1 cells, a human monocytic cell line, were cultured in RPMI‐1640 medium supplemented with 10% fetal bovine serum and 20 mm Glutamine (200 mm solution, Gibco, Life Technologies Corporation, USA) at 37°C in a humidified atmosphere containing 5% CO_2_. To differentiate THP‐1 monocytes into macrophages, cells were treated with 40 ng mL^−1^ human macrophage colony‐stimulating factor (MCSF; Miltenyi Biotec, Germany) for 10 days before further experiments.

### 4.14 Uptake Assay

A total of 5 × 10^4^ differentiated THP‐1 macrophages were seeded into a 96‐well culture plate and let attach and reach confluence for 48 h. Next, the cells were incubated with 1 × 10^9^ MVs labeled with DiI in a final volume of 200 µL of fresh RPMI‐1640 medium. The cells were maintained at 37°C in a humidified atmosphere containing 5% CO_2_. The uptake of MVs was evaluated at multiple time points (0.5, 2, 4, and 24 h) post‐incubation by flow cytometry and confocal laser scanning microscopy.

For flow cytometry analyses, at each time point the cells were detached from the wells by a vigorous pipetting. Then, the cells were centrifuged at 400 g for 5 min, 100 µL supernatant was removed and the cells were dispersed in fresh RPMI‐1640 (without supplements). The number of DiI‐positive cells were measured using a CytoFlex SRT (Beckman Coulter). Data were collected from a minimum of 5 000 events per sample, using a side scatter (SSC) threshold set at 1 500 to filter out noise. The percentage of DiI‐positive macrophages was then analyzed using CytExpert software (Beckman Coulter, USA).

For CLSM imaging, at each time point the cell culture supernatant was removed and THP‐1 macrophages were labeled 15 min at 37°C with DiD and DAPI to visualize cell membranes and nuclei, respectively. After removing the stain solutions, the adherent cells were gently washed with PBS and fixed with paraformaldehyde 3,7%. After washing with PBS, cells were saved for imaging with a confocal laser scanning microscope (FV3000, Olympus, Japan) with appropriate filters for DiI (570–670 nm), DiD (643–708 nm) and DAPI (430–470 nm). Lasers with excitation wavelengths of 561 nm for the DiI channel, 640 nm for the DiD channel, and 405 nm for the DAPI channel were configured for visualization. Image analysis was performed using ImageJ v1.53 and Imaris 9.9.0 software (Oxford Instruments, UK).

### 4.15 Caco‐2 Cell Culture and Maestro Z Impedance Measurements

Before plating the cells, 100 µL of Dulbecco's Modified Eagle Medium supplemented with 2% fetal bovine serum was added to each well of CytoView‐Z 96‐well electrode plates (Axion Biosystems, Atlanta, GA, USA). The CytoView‐Z plates were then connected to the Maestro Z instrument to establish a baseline measurement of the impedance electrodes. Approximately 50 000 Caco‐2 cells per well were added to the plates and allowed to sit at room temperature for one hour to ensure uniform distribution across the wells. Following this, the plates containing Caco‐2 cells were placed in the Maestro Z for up to 24 h at 37°C with 5% CO_2_, allowing the cells to adhere and form a confluent monolayer, as indicated by impedance measurements. Maestro Z monitored the impedance of the monolayer, which is analogous to transepithelial electrical resistance. Resistance was recorded at frequencies of 41.5–1 kHz, to comprehensively evaluate cell coverage and the integrity of tight junctions, respectively [59]. Resistance measurements were continuously taken for 48 h post‐treatment at 37°C with 5% CO_2_ in a humidified environment using the Maestro Z instrument. All plates included controls with media only and MV samples. Raw resistance values for each MV‐treated well were normalized against medium controls and against values recorded just before MV treatment. Measurements were performed in quadruplicate for each condition. Data analysis was performed using Axis Z software (Axion Biosystems, Atlanta, GA, USA) [59].

### 4.16 Lipoteichoic acid (LTA) Dot Blot Analysis

Since *L. casei* BL23 MVs are known to contain LTA, we used dot blot to confirm the isolation of MVs by SEC. For each sample type (bacteria, UC pellet, MVs, and protein fraction), an equal amount of protein (5.12 µg) was loaded onto a nitrocellulose membrane in a dot blot format. The membrane was blocked with blocking buffer (1X Tris‐buffered saline, 0.1% Tween‐20 with 5% w/v nonfat dry milk) for 1 h at room temperature, followed by incubation overnight at 4°C with anti‐LTA (G43J monoclonal mouse antibody, ThermoFisher Scientific, MA1‐7402) diluted in blocking buffer (1:500). After incubation, the membrane was washed three times with Tris‐buffered saline for 10 min each wash. The membrane was then incubated with an HRP‐conjugated secondary antibody (Anti‐mouse IgG HRP‐linked Antibody, Cell Signaling, 7076S) diluted in blocking buffer (1:2000) for 1 h at room temperature. After three washes with Tris‐buffered saline, LTA detection was performed using an ECL detection kit (BioRad Laboratories, Inc.) according to the manufacturer's instructions. The membrane was scanned using Li‐Cor 3600 C‐Digit Blot Scanner with the software Li‐COR Acquisition V.2.2.

### 4.17 Caenorhabditis Elegans and Bacterial Growth Conditions

*Pseudomonas aeruginosa* PA14 and *E. coli* OP50 were grown in LB broth at 37°C overnight at 160 rpm. The wild‐type *Caenorhabditis elegans* (*C. elegans*) Bristol strain N2 was maintained at 20°C with *E. coli* OP50 as a food source on nematode growth medium agar plates (NGM) according to standard procedures [69, 70]. Four gravid N2 were transferred onto each of three lawns of *E. coli* OP50 and NGM plates were incubated at 20°C until the L4‐stage was reached.

### 4.18 *C. elegans* Fast‐Killing Assay

The fast‐killing assay was performed on PGS agar plates. Briefly, 50 µL of each overnight *E. coli* OP50 and *P. aeruginosa* PA14 culture was centrifuged at 9500 g for 5 min to pellet the bacteria, and the pellets were resuspended in either 50 µL of MVs (1 × 10^10^) or 50 µL of PBS. The resuspended cultures were pipetted onto 3.5 cm PGS agar plates, and incubated upside down at 37°C for 24 h to form a confluent lawn. The plates were then transferred to 23°C and incubated for an additional 24 h [61]. Forty L4‐stage N2 worms were transferred to each OP50 or PA14 PGS plate. After 4 and 24 h, worms were classified to dead or alive, using a touch movement test. The percentage of living worms was calculated for each plate of each condition. The assay was performed with three independent biological replicates. Two of these included two technical replicates, and the experiments were run by two different experimenters.

### 4.19 Statistical Analysis

All data are presented as mean ± standard deviation, with the number of independent experiments (n) indicated in each figure. Each measurement was conducted in at least three independent replicates. Statistical analysis was performed using RStudio, employing One‐way ANOVA followed by Tukey's post‐hoc test to compare mean values across different groups, unless specified. Significant p‐values are denoted by ^*^ for *p* < 0.05, ^**^ for *p* < 0.005, ^***^ for *p* < 0.001 and ^****^ for *p* < 0.0001.

## Author Contributions

C.L.D., A.P.D.R., and G.F. conceived and designed the study. C.L.D., L.P.J., L.S., and M.D. performed the experiments. J.M. contributed to the primary cell isolation process and to the qPCR experiments. H.D. contributed to the impedance measurements. C.L.D., L.P.J., and L.S. acquired the data. C.L.D and L.P.J. analyzed and interpreted the data. M.L. and E.Z. contributed to the experimental design of tests on *C. elegans*, provided reagents and methodological training. P.A. acquired transmission electron microscopy (TEM) images. C.L.D., G.F., and O.E.P. drafted or revised the article. All the coauthors have read and confirmed the submitted manuscript.

## Conflicts of Interest

The authors declare no conflicts of interest.

## Supporting information




**Supporting Information**: adhm70580‐sup‐0001‐SuppMat.docx

**Supporting Information**: adhm70580‐sup‐0002‐VideoS1.mp4

## Data Availability

The original contributions presented in this study are included in the article and/or Supplementary Material. Further inquiries can be directed to the corresponding author.
