# Supplementary material for: Membrane Vesicles from Lacticaseibacillus Casei BL23 Exhibit Antimicrobial Activity Against Escherichia coli and Immunostimulatory Effects on Human Peripheral Blood Mononuclear Cells
Source: Adv Healthc Mater. 2025 Dec 5;15(16):e00548. doi: 10.1002/adhm.202500548 (PMC13107934; doi:10.1002/adhm.202500548)
Supplement: Supplementary file 1 — Supporting Information: adhm70580‐sup‐0001‐SuppMat.docx [file ADHM-15-0-s002.docx]

Membrane Vesicles from *Lacticaseibacillus casei* BL23 Exhibit Antimicrobial Activity against *Escherichia coli* and Immunostimulatory Effects on Human Peripheral Blood Mononuclear Cells

Cecilia L. D’Antoni, Leila Pourtalebi Jahromi, Lorenzo Sana, Ana Paula Domínguez Rubio, Maja Dorfner, Jennifer Munkert, Heike Danzer, Philipp Arnold, Mikhail Lebedev, Esther Zanin, Oscar E. Pérez, Gregor Fuhrmann*

Cecilia L. D’Antoni, Ana Paula Domínguez Rubio, Oscar E. Pérez.

Departamento de Química Biológica de la Facultad de Ciencias Exactas y Naturales Universidad de Buenos Aires

Buenos Aires C1428EGA, Argentina.

Cecilia L. D’Antoni, Ana Paula Domínguez Rubio, Oscar E. Pérez

Instituto de Química Biológica de la Facultad de Ciencias Exactas y Naturales

Universidad de Buenos Aires, Consejo Nacional de Investigaciones Científicas y Técnicas

Buenos Aires C1428EGA, Argentina.

Cecilia L. D’Antoni, Leila Pourtalebi Jahromi, Lorenzo Sana, Maja Dorfner, Jennifer Munkert, Gregor Fuhrmann*

Department of Biology, Pharmaceutical Biology

Friedrich-Alexander-Universität Erlangen-Nürnberg (FAU)

91058 Erlangen, Germany.
E-mail: [gregor.fuhrmann@fau.de](mailto:gregor.fuhrmann@fau.de)

ORCID IDs: Gregor Fuhrmann 0000-0002-6688-5126

Cecilia L. D’Antoni 0000-0002-7352-1857

Gregor Fuhrmann

FAU NeW

Friedrich-Alexander-Universität Erlangen-Nürnberg (FAU)

Erlangen 91058, Germany

Heike Danzer

Deutsches Zentrum Immuntherapie (DZI) and Department of Internal Medicine 3, Rheumatology and Immunology

Friedrich-Alexander-Universiät Erlangen-Nürnberg (FAU) and Universitätsklinikum Erlangen

91054, Erlangen, Germany.

Philipp Arnold
Institute of Anatomy, Functional and Clinical Anatomy
Friedrich-Alexander-University Erlangen-Nürnberg (FAU)
91054 Erlangen, Germany

[Mikhail Lebedev](https://www.cell.com/cell-reports/fulltext/S2211-1247(23)01087-2?uuid=uuid%3A43ec53f7-ca98-4f74-b124-3eff21e79eaa), Esther Zanin

Department of Biology

Friedrich-Alexander-Universität Erlangen-Nürnberg (FAU)

91058 Erlangen, Germany.

**Supporting Information**

**Table S1.** List of primers utilized for assessing cytokine expression in peripheral blood mononuclear cells (PBMCs) through quantitative real-time PCR (qPCR).

| Primers name | Sequence | Tm ºC | Reference |
| --- | --- | --- | --- |
| 18S RNA_hu_mu S1489_fw | AGGTCTGTGATGCCCTTAGA | 57.3 | ^[47]^ |
| 18S RNA_hu_mu A1579_rev | GAATGGGGTTCA ACGGGTTA | 57.3 |  |
| IL1ß_hu_32_fw | GGCTGCTCTGGGATTCTCTT | 59.4 | ^[47]^ |
| IL1ß_hu_151_rev | AGTCATCCTCATTGCCACTGTAA | 58.9 |  |
| IL6_hu_239_fw | ACATCCTCGACGGCATCTCA | 59.4 | ^[47]^ |
| IL6_hu_403_rev | TCACCAGGCAAGTCTCCTCATT | 60.3 |  |


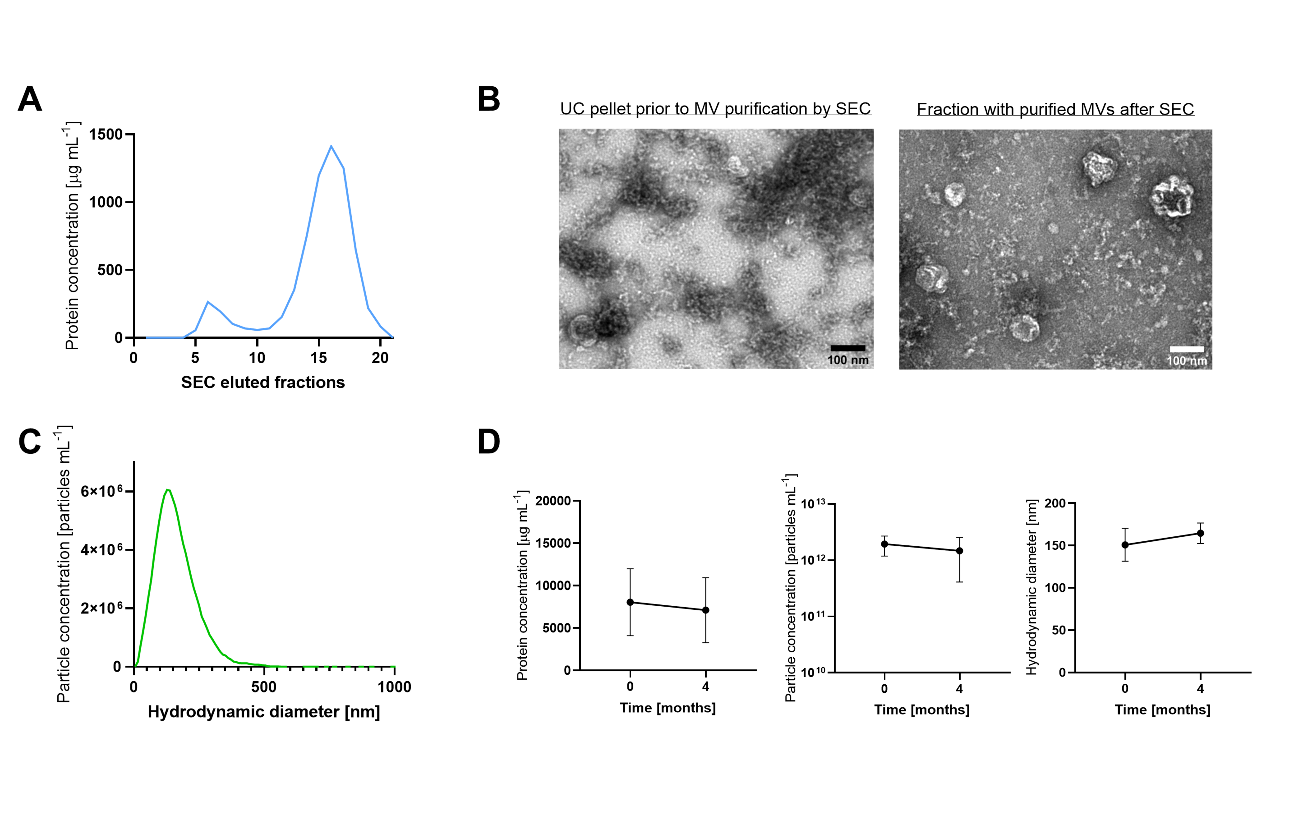


**Figure S1. MV characterization.** **A.** Separation of *L. casei* BL23 MVs from soluble proteins by Size Exclusion Chromatography. Fraction 6 enriched in EVs (271 µg mL^-1^) and Fraction 16 enriched in soluble proteins (n=12). **B.** Representative TEM images with a 100 nm scale of the UC pellet before MV purification (left) and fraction 6 containing EVs after SEC purification (right). **C.** Representative size distribution curve on a fraction of SEC enriched in EVs (Fraction 6) obtained by Nanoparticle Tracking Analysis (n=4).

**D.** Protein concentration, particle concentration, and hydrodynamic diameter of *L. casei* MVs before and after storage at 4°C for 4 months in PBS (n=5). Mean ± SD, p > 0.05; Student’s t-test. Mean ± SD, p > 0.05; Student’s t-test.


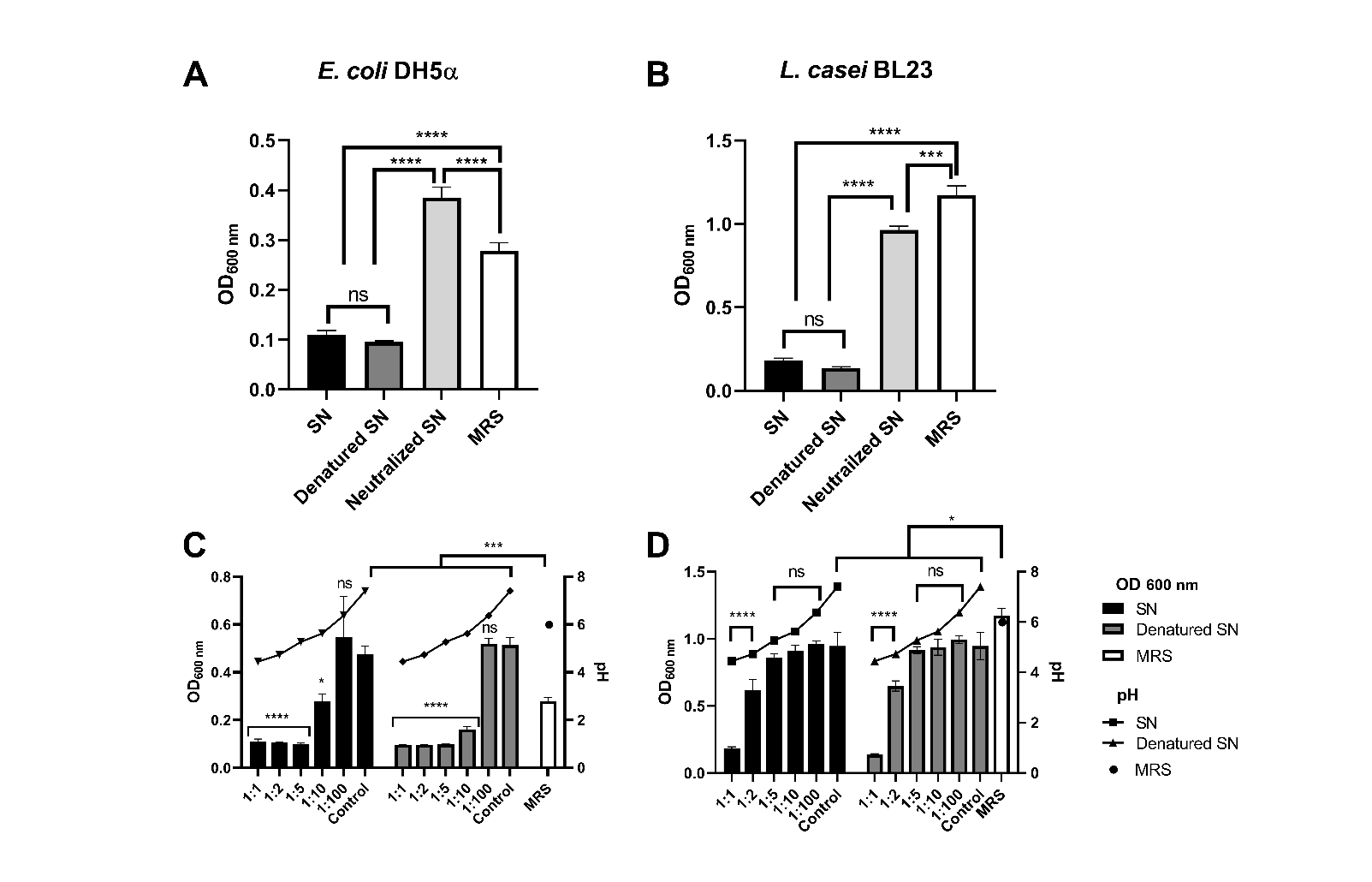


**Figure S2.** **A.** Optical density (OD) of *E. coli* culture incubated for 15 h with *L. casei* BL23 ultracentrifugation supernatant (SN), denatured SN and neutralized SN. MRS is shown as a control. Bars represent mean value and standard deviation (n = 3). Asterisks indicate significant difference compared to the control (*p* < 0.05; one-way ANOVA followed by Tukey comparisons). **B.** Optical density (OD) of *L. casei* culture incubated for 20 h with *L. casei* BL23 ultracentrifugation supernatant (SN), denatured SN and neutralized SN. MRS is shown as a control. Bars represent mean value and standard deviation (n = 3). Asterisks indicate significant difference compared to the control (*p* < 0.05; one-way ANOVA followed by Tukey comparisons). **C.** Optical density (OD) of *E. coli* culture incubated for 15 h with different dilutions *L. casei* BL23 ultracentrifugation supernatant (SN) and denatured SN obtained from *L. casei* grown in MRS. Bars represent mean value and standard deviation (n = 3). Asterisks indicate significant difference compared to the control (*p* < 0.05; one-way ANOVA followed by Tukey comparisons). **D.** Optical density (OD) of *L. casei* culture incubated for 20 h with different dilutions of *L. casei* BL23 ultracentrifugation supernatant (SN) and denatured SN obtained from *L. casei* grown in MRS. Bars represent mean value and standard deviation (n = 3). Asterisks indicate significant difference compared to the control (*p* < 0.05; one-way ANOVA followed by Tukey comparisons).


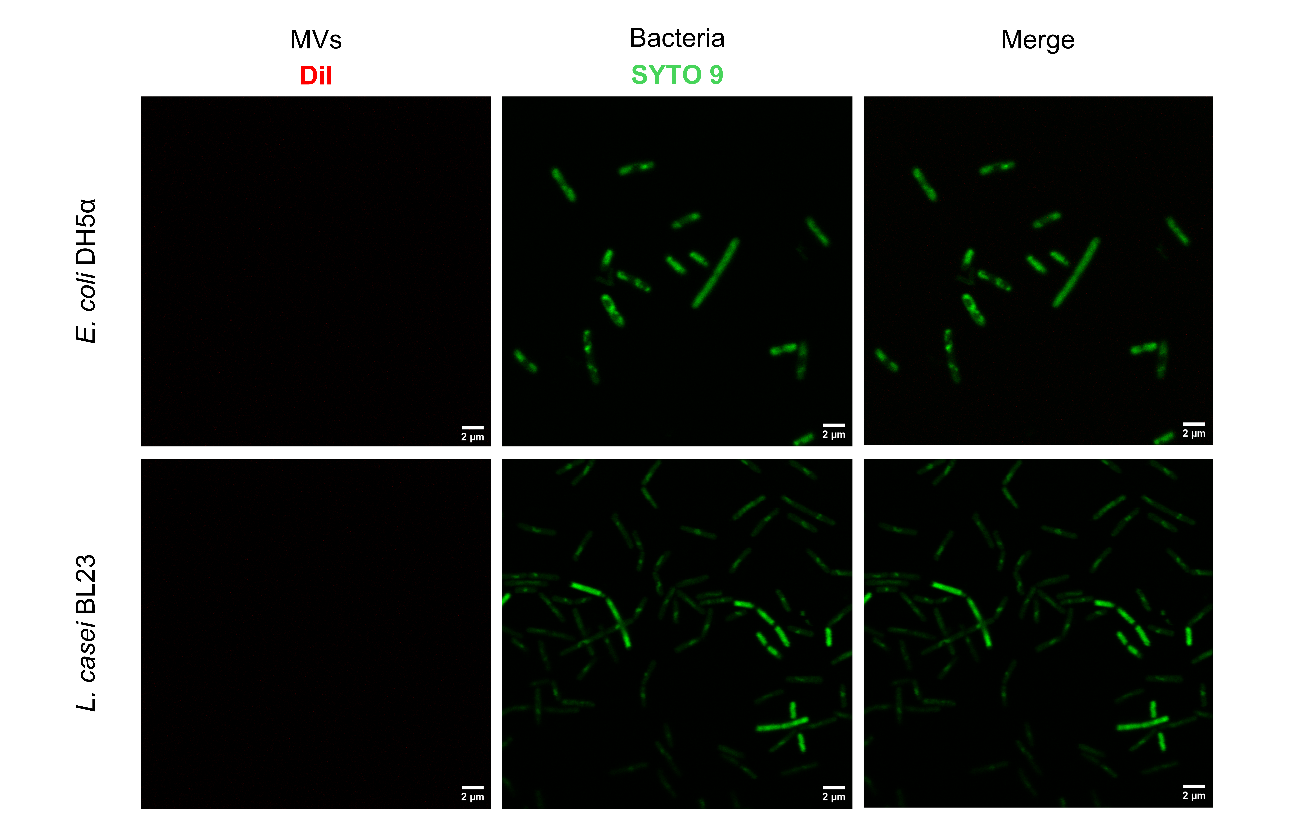


**Figure S3**. Representative confocal fluorescence images of *E. coli* and *L. casei* incubated with PBS for 24 h (negative control, without MVs). Images were captured using consistent laser settings at 561 nm and 488 nm. Scale bars indicate 2 μm.


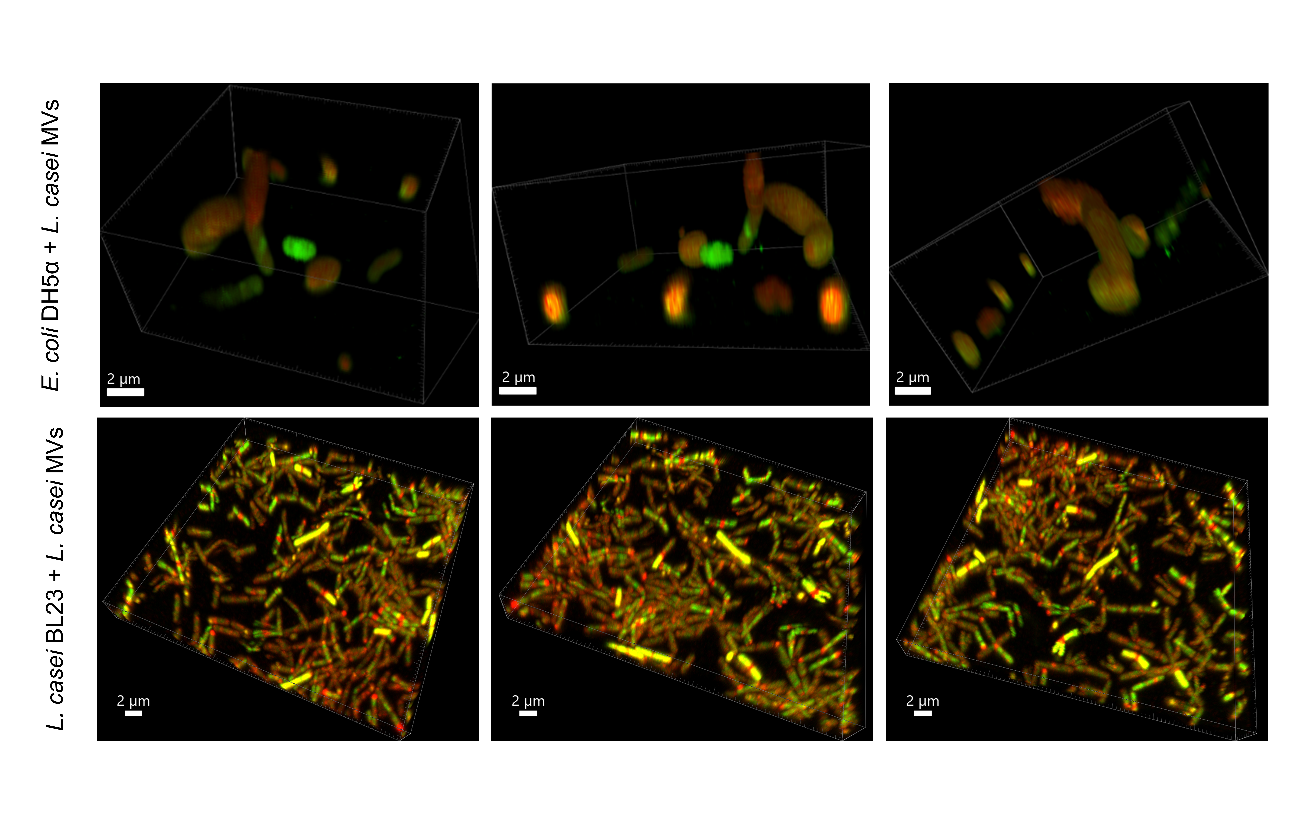


**Figure S4.** Different angles of visualization of 3D images of *E. coli*and *L. casei* incubated with *L. casei* MVs were obtained using confocal microscopy, featuring a slice thickness of 0.38 μm across 10 to 19 slices. The images were processed in Imaris 9.9.0 software (Oxford Instruments, UK) to enhance the visibility of 3D structures, by background subtraction.

**
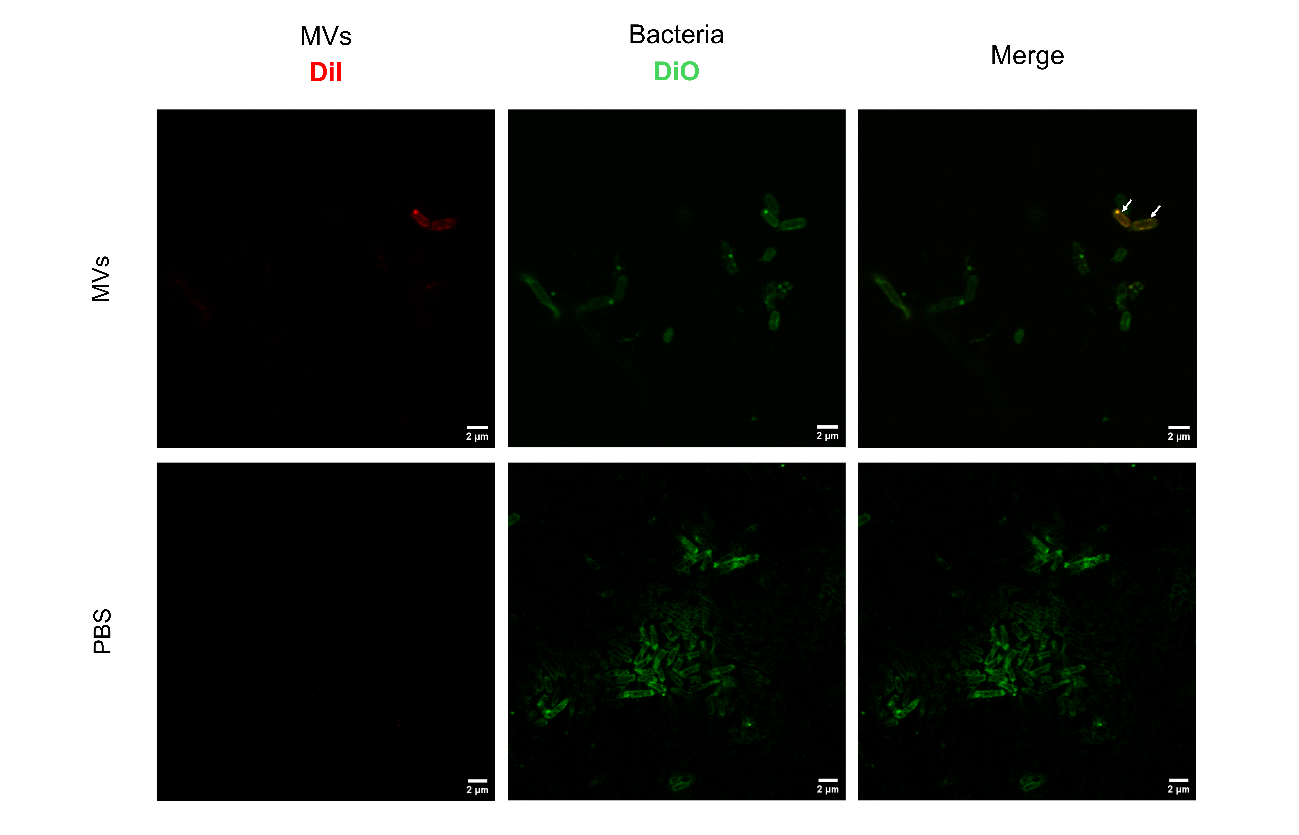
**

**Figure S5.** Representative confocal fluorescence images of *E. coli* incubated with MVs and PBS (control). Bacteria were incubated with fluorescently (DiI)-labelled MVs (10^10^ mL^-1^) or PBS for 30 minutes. Images were captured using consistent laser settings at 561 nm and 488 nm. Colocalization is indicated by a white arrow. Scale bars indicate 2 μm.


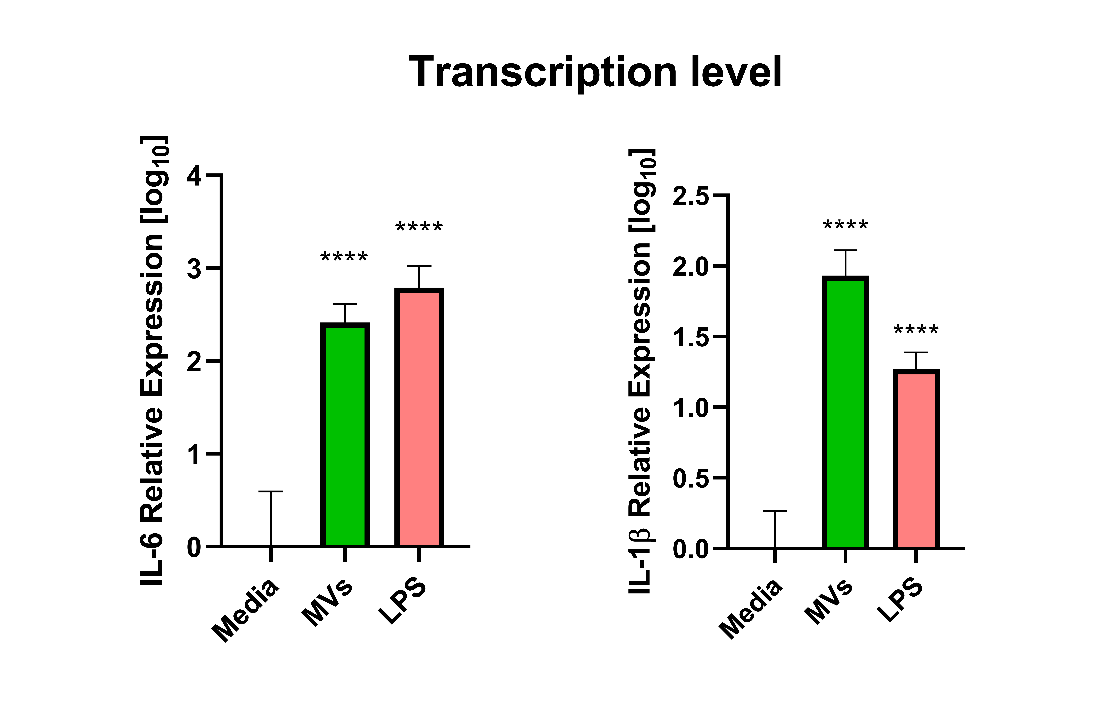


**Figure S6.** mRNA Levels of Cytokines in MV-Treated PBMCs Analyzed by qPCR. Bars represent cytokine gene expression relative to that of 18S rRNA (reference gene) for each treatment condition. The results are presented as log10-transformed values of the RQ (relative quantification value). Mean ± SD, One-way ANOVA followed by Tukey *post-hoc* test (n=3). Asterisks indicate significant difference compared to the control (*p* < 0.05; one-way ANOVA followed by Tukey comparisons).





**Figure S7.** The dot blot image for detection of lipoteichoic acid (LTA) in *L. casei* and its MVs, as well as the controls.


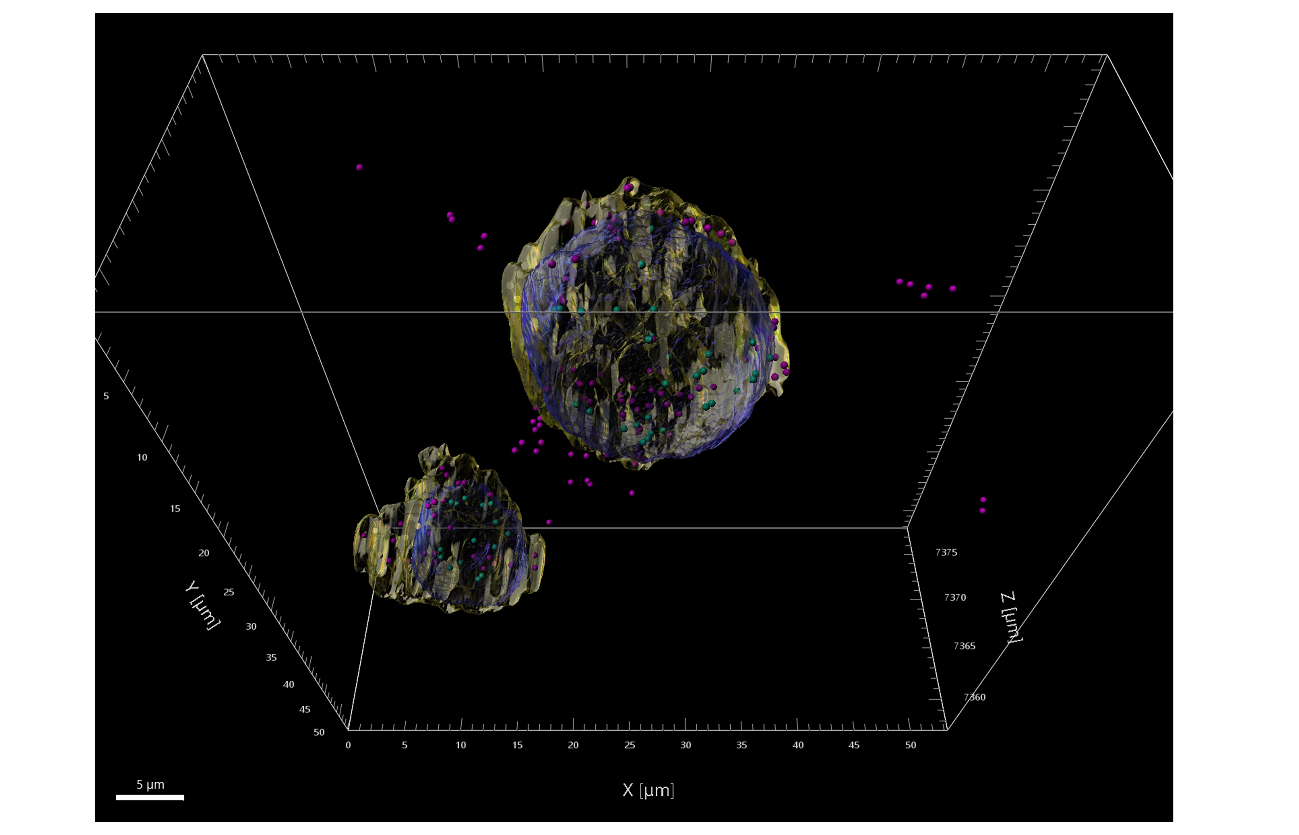


**Video S1.** The video presents two macrophages following a 24-hour incubation with *L. casei* MVs. Small dots represent MVs. Purple MVs denote MVs located within the cytoplasm and outside the macrophages. The cyan dots indicate the presence of MVs within the nucleus. Yellow represents the cell membrane of the macrophage. The 3D rendering was created using Imaris 9.9.0 software (Oxford Instruments, UK).
